# ﻿Phylogenetic analysis suggests early divergence followed by convergent morphological evolution in the *Silene* sections *Odontopetalae* and *Sordidae* (Caryophyllaceae)

**DOI:** 10.3897/phytokeys.265.165998

**Published:** 2025-10-24

**Authors:** Zeynep Toprak

**Affiliations:** 1 Department of Molecular Biology and Genetics, Faculty of Sciences, University of Dicle, Diyarbakır, Turkiye University of Dicle Diyarbakır Turkiye

**Keywords:** Convergent evolution, phylogeny, *
Silene
*, *Silene odontopetala*, *Silene sordida*, StarBeast3

## Abstract

The genus *Silene* presents significant taxonomic challenges, particularly for groups such as S. sect. Odontopetalae and the monotypic S. sect. Sordidae. This study investigates the evolutionary relationship between the narrowly endemic *Silene sordida* and the widespread *S. odontopetala* to resolve these ambiguities. Using a multispecies coalescent framework with five genetic markers and expanded taxon sampling, the species tree and divergence times were estimated. The results revealed a moderately supported sister relationship between *S. sordida* and S. sect. Odontopetalae, with their divergence estimated at approximately 5.5 million years ago, following the Messinian salinity crisis. Despite their profound morphological and ecological differences, the results suggest a shared evolutionary origin. This study underscores the limitations of morphology-based classification in *Silene* and highlights the critical roles of ecological divergence, historical biogeography, and convergent evolution in shaping the genus’s diversity. The results provide a clearer understanding of the evolutionary processes driving diversification in these complex lineages.

## ﻿Introduction

*Silene* L. (Caryophyllaceae) comprises approximately 870 species, predominantly distributed across temperate and alpine regions of the Northern Hemisphere (Oxelman and Lidén 1995; Eggens et al. 2007; Jafari et al. 2020). The genus is most diverse in the eastern Mediterranean Basin and southwestern Asia (Ghazanfar 1983; Jeanmonod 1984; Greuter 1995; Oxelman et al. 2001; Yıldız 2024). Turkiye and Greece, home to many endemic *Silene* species, rank among the regions richest in *Silene* diversity (Coode and Cullen 1967; Meikle 1977; Greuter 1997; Yıldız and Çırpıcı 2013; Aydin et al. 2014a).

Inferring phylogenies within *Silene* is challenging and often conflicts with morphology-based classifications (Oxelman et al. 1997; Popp et al. 2005; Đurović et al. 2017; Naciri et al. 2017). While several global taxonomic revisions have been proposed (Otth 1824; Rohrbach 1869; Chowdhuri 1957; Lazkov 2003; Jafari et al. 2020), key morphological traits used for classification—such as life habit, style number, petal shape, seed and pollen structure, indumentum, and anthophore length—often exhibit significant homoplasy. This complicates both generic and infrageneric classification. Molecular studies have addressed several uncertainties at subgeneric and sectional levels (Popp and Oxelman 2004, 2007; Frajman et al. 2009; Petri and Oxelman 2011; Rautenberg et al. 2012; Aydin et al. 2014b; Moiloa et al. 2021). The most recent global revision of the genus (Jafari et al. 2020) recognized 33 sections within *Silene*, grouped into three well-supported subgenera: S. subg. Silene, S. subg. Behenantha (Otth) Endl., and S. subg. Lychnis (L.) Greuter. Other research has focused on delineating section boundaries (Gholipour and Sheidai 2010a, 2010b; Rautenberg et al. 2010; Naciri et al. 2017; Eggens et al. 2020), as well as exploring relationships within species complexes (Peruzzi and Carta 2013; Toprak et al. 2016; Du Pasquier et al. 2017). Nevertheless, the genus remains taxonomically complex, with unresolved ambiguities at lower taxonomic levels and unclear phylogenetic relationships among groups such as S. sect. Odontopetalae Chowdhuri and S. sect. Sordidae K. Yıldız & Toprak.

*Silene odontopetala* Fenzl, the type of S. sect. Odontopetalae, is a widely distributed perennial found in high-altitude regions of southwestern (e.g., Iran, Turkiye, Iraq, Lebanon, Syria, Sinai, and the Caucasus) and Central Asia (e.g., Uzbekistan) (GBIF 2025; POWO 2025). Phylogenetically, *S. odontopetala* and several closely related species, including *S. joerstadii* Wendelbo and *S. auriculata* Sibthorp & Smith, share strong genetic ties with a number of Asian taxa classified within S. sect. Physolychnis (Benth.) Bocquet, a group significantly shaped by both ancient and recent hybridization events that have influenced its evolutionary history (Petri and Oxelman 2011; Petri et al. 2013; Frajman et al. 2018). In contrast, *Silene sordida* Huber-Morath & Reese is an annual species with a restricted range, endemic to Anatolia and found only in Muğla Province, southwestern Turkiye (Coode and Cullen 1967). Despite being studied in several phylogenetic analyses (Erixon and Oxelman 2008; Jafari et al. 2020; Toprak and Yıldız 2022), the taxonomy of *S. sordida* remains poorly understood. Historically, it was assigned to S. sect. Atocion Otth (Coode and Cullen 1967), a group with an uncertain phylogenetic position within *Silene*. However, molecular and morphological analyses later demonstrated this classification to be erroneous, and the species was reassigned to the newly described monotypic section S. sect. Sordidae (Toprak and Yıldız 2022). Taxonomically, both S. sect. Sordidae and S. sect. Odontopetalae are classified within S. subg. Behenantha, the clade encompassing 18 of the 33 recently revised sections by Jafari et al. (2020).

The evolutionary history of *S. sordida* and *S. odontopetala* is largely unknown. Morphologically, these two species are highly divergent in terms of their habitat, plant size, branching pattern, floral characteristics, seed structure, and life cycle, among other traits. Nevertheless, recent molecular studies (Jafari et al. 2020; Toprak and Yıldız 2022) have reported close relationships between *S. sordida* and members of S. sect. Odontopetalae, raising the possibility of an ancient or recent hybridization event between the taxa. However, studies including both species have been limited to independent gene tree analyses based on very few loci, with sequencing conducted on a small number of samples of *S. odontopetala*.

Recent developments in phylogenetic methods, particularly those based on the multispecies coalescent (MSC) model (e.g., Nordborg 2003; Degnan and Salter 2005; Liu et al. 2008; Heled and Drummond 2010; Jones et al. 2015), have greatly enhanced our ability to study species relationships. Consequently, a comprehensive phylogenetic analysis that incorporates advanced species tree approaches (e.g., Pamilo and Nei 1988; Maddison 1997; Kubatko et al. 2009; Zhang et al. 2018), accounting for both topological incongruences and evolutionary timescales, may provide new insights into complex relationships such as those among the sections *Odontopetalae* and *Sordidae* (Luo et al. 2023). In particular, sufficient sampling of *S. odontopetala*, *S. sordida*, and members of S. sect. Cryptoneurae Aydın & Oxelman and S. sect. Physolychnis, their potential closest relatives, could help resolve key uncertainties. Moreover, divergence time estimates could further enrich our understanding of their historical dynamics (dos Reis et al. 2016). However, estimating divergence dates for *Silene* remains challenging due to complications such as the lack of informative fossils, limited phylogenetic resolution, and variations in substitution rates. Despite methodological challenges, previous studies utilized a 34–45 Ma (million years ago) caryophyllaceous fossil (Jordan and Macphail 2003) for calibration. This allowed them to estimate the root age of *Silene* at approximately 11.6 Ma (95% highest posterior density [HPD] interval: 9.177–12.17; Frajman et al. 2009; Rautenberg et al. 2010, 2012). This mid-Miocene (ca. 5.3–23 Ma) divergence serves as a key calibration point for evaluating the dispersal and colonization history of some major lineages within the genus (Moiloa et al. 2021).

This study aims to elucidate the phylogenetic relationships between *S. sordida* and *S. odontopetala*. To achieve this, taxon sampling was expanded to cover a wider distribution range of *S. odontopetala* in Turkiye, including two samples from Iran, and additional samples of *S. sordida* together with its Mediterranean and South Aegean allies from S. sect. Cryptoneurae were incorporated. Sequence data from five loci were generated and combined with previously published data for the corresponding taxa. Using an MSC-based Bayesian approach to species tree estimation and incorporating the available dating information, the relationships of the focal taxa, *S. sordida*, *S. odontopetala*, and S. sect. Cryptoneurae were investigated.

## ﻿Materials and methods

### ﻿Taxon sampling

The plant material used in this study includes *Silene* species from the following sections, as classified by Jafari et al. (2020) and Toprak and Yıldız (2022): *Odontopetalae*, *Sordidae*, *Cryptoneurae*, *Physolychnis*, *Siphonomorpha* Otth, and *Auriculatae* (Boiss.) Schischk. Based on Jafari et al. (2020), we focused on sampling species from sections *Odontopetalae*, *Physolychnis*, and *Cryptoneurae*, which could potentially be sister groups to *Sordidae*. To represent S. subg. Silene, *S. italica* (L.) Pers., *S. nemoralis* Waldst. & Kit., and *S. schafta* J.G.Gmel. ex Hohen., belonging to sections *Siphonomorpha* and *Auriculatae*, respectively, were included.

In total, 16 *Silene* species sampled from a wide geographic range, including Alaska, Greenland, Russia, China, and Korea, were investigated. Most of the plant material was obtained from herbarium specimens collected during field surveys conducted in Turkiye between 2005 and 2020. These materials are preserved in the herbaria GB, UPS, and CBAH. Herbarium codes follow Index Herbariorum (Thiers 2023). For species occurring outside Turkiye (e.g., Russia, Alaska, Iran, Afghanistan), sequence data were sourced from GenBank. Details of the specimens are provided in Suppl. material 1: table S1. Notably, 11 samples were sequenced for the first time, including five of *S. odontopetala*, two of *S. sordida*, three of *S. cryptoneura*, and one of *S. ertekinii*, across five loci.

### ﻿Molecular data

In total, 216 sequences (GenBank IDs are provided in Suppl. material 1: table S1), including 24 for *S. sordida* and 33 for *S. odontopetala*, were used. Sequence data were generated from the nuclear DNA (nrDNA) regions *RPA2* (47 sequences), *RPB2* (46 sequences), *EST04* (26 sequences), *ITS* (49 sequences), and the chloroplast DNA (cpDNA) region *rps16* (48 sequences). These markers have been commonly used in recent phylogenetic studies of the Caryophyllaceae family (Fior et al. 2006) and the genus *Silene* (Oxelman and Lidén 1995; Popp and Oxelman 2004; Popp et al. 2005; Petri et al. 2013; Aydin et al. 2014b; Toprak et al. 2016; Naciri et al. 2017; Jafari et al. 2020; Moiloa et al. 2021). The final dataset comprised 216 sequences from 16 species, with *S. italica*, *S. nemoralis*, and *S. schafta* used as outgroups, as they are distantly related to the remaining taxa.

Total DNA was extracted from 25 mg of dried leaf material using a tissue lyser, following the DNeasy Plant Mini Kit (Qiagen, Germany) protocol. Considering the manufacturer’s recommendations, the final PCR reaction volume was adjusted to 25 μl, containing 8 μl of kit components, 2 μl of each primer, 1 μl of the sample, and 12 μl of nuclease-free water. Amplifications of the *rps16* and *ITS* regions were performed using the Vivantis DNA amplification kit (Vivantis, Lithuania) on a TC-512 PCR instrument (Techne, Staffordshire, UK) with custom programs. The remaining loci were amplified using the iTaq Universal Supermix kit (Bio-Rad, USA) on a CFX Connect Real-Time PCR machine (Bio-Rad, USA). Primer sequences and PCR programs are listed in Suppl. material 1: table S2. All PCR products were analysed on a 1.25% agarose gel to verify approximate amplicon sizes. Positive samples were purified using the QIAquick PCR Purification Kit (Qiagen, Germany) according to the manufacturer’s guidelines. Cleaned products were outsourced to Atlas Biotechnologies (Ankara, Turkiye) for cycle sequencing.

### ﻿Phylogenetic analyses

Contigs were visually inspected and edited using Geneious Prime 2022.1.1 (Biomatters, USA). Sequences obtained from GenBank were manually added to the datasets. The contigs were aligned using the MUSCLE alignment tool in Geneious Prime 2022.1.1, with alignment positions visually checked and adjusted as needed.

Parsimony statistics for each dataset were calculated using PAUP* 4.0a.169 (Swofford 2002, 2021). All loci, including the cpDNA *rps16* locus, were tested for recombination detection using RDP v4.101 (Martin et al. 2015) under the methods RDP, GENECONV, Chimaera, MaxChi, and 3Seq at a significance level of 0.1. Substitution models were selected based on the Akaike Information Criterion (AICc) using the automated model selection function implemented in PAUP* v4.0a.169.

Species and gene trees were estimated simultaneously using StarBEAST3 v1.2.1 (Douglas et al. 2022) in BEAST v2.7.7 (Bouckaert et al. 2014, 2019). StarBEAST3 is a Bayesian software for species tree inference under the MSC model, jointly estimating species and gene trees while accounting for gene tree–species tree discordance. It uses adaptive parallelized MCMC algorithms to improve computational efficiency. Input files were prepared in BEAUti 2.7.7, and several preliminary analyses were conducted with alternative settings for fixed-locus rates, prior distributions, and clock models. All StarBEAST3 analyses were also run with data files sampled from priors. The analyses were performed on the CIPRES portal (Miller et al. 2010).

Species trees were estimated under both relaxed and strict clock models. The ploidy level for the *rps16* region was set to half that of the nuclear loci, as the plants are hermaphroditic. One set of analyses was conducted in which the relative clock rate of each partition was checked against the *RPA2* locus (since it has the largest fraction of segregating sites) with a mean mutation rate of 1.0. However, the final species trees were generated independently under both clock models, where each locus had its own rate estimated.

In the analyses performed under each clock model separately, gene tree clock rates followed a lognormal prior with standard settings. A Yule prior was applied to the species tree, with the speciation rate drawn from a lognormal prior (mean = 1.0, standard deviation = 1.25). Additionally, a lognormal prior (mean = −6.0, SD = 2.0) was used for the mean population size. For the species tree generated under the strict clock model, the clock rate was assigned a normal prior with default values. In the species tree estimated under the relaxed clock model, the clock rate followed a lognormal prior (mean = 1.0, standard deviation = 1.25) with the mean parameter set in real space.

The species tree was calibrated by defining the prior age of the most recent common ancestor (MRCA) of S. subg. Silene and S. subg. Behenantha, with a root height set to a mean of 11.6 million years as reported in previous studies (Frajman et al. 2009; Rautenberg et al. 2012; Moiloa et al. 2021), under a normal distribution and a standard deviation of 0.8. This was done by defining an MRCA prior encompassing all samples belonging to both subgenera. Three independent analyses were run for 300 million generations each, with samples taken every 20,000 generations and discarding the first 25% as burn-in. Mixing and convergence were assessed using Tracer v1.7.1 (Rambaut et al. 2018). Maximum clade credibility trees were generated using TreeAnnotator v2.7.7 (Bouckaert et al. 2014, 2019), after discarding 25% of the trees as burn-in. The final species and gene trees were visualized with FigTree v1.4.4 (Rambaut 2018). To illustrate the compatibility of the gene trees with the species tree estimated under the strict clock model, tree files were uploaded to UglyTrees (Douglas 2021) and processed accordingly.

To interpret node conflicts observed in the species trees, ASTRAL-III v5.7.8 (Mirarab and Warnow 2015) was used to estimate quartet scores (Sayyari and Mirarab 2016) for the tree branches. This method calculates the fraction of sampled gene-tree quartets that match the species-tree topology, using unrooted gene trees of the analysed loci (Sayyari et al. 2018; Zhang et al. 2018). As input for ASTRAL-III, maximum likelihood gene trees for the five loci were estimated via RAxML (Stamatakis 2014) as implemented in Geneious Prime 2022.1.1, using rapid bootstrapping and the search for the best-scoring ML tree option. The resulting gene trees were then processed in ASTRAL-III in comparison with a reference species tree (each species tree estimated under different clock models using StarBEAST3), following the documentation at https://github.com/smirarab/ASTRAL. The analysis was run with the “-q reference_species.tre” flag to score the existing tree, the full set of gene trees (-i all_gene_trees.tre), and the required species map (-a 16_species_map.txt). The branch annotation level was set to “-t 2,” instructing ASTRAL to output raw fractional quartet support values (q1, q2, and q3) for every internal split, thereby directly quantifying the proportion of gene tree agreement for the reference topology relative to the two alternatives.

To further compare the estimated species trees under different clock models statistically, a model selection test was performed based on marginal likelihoods (MLE) using the nested-sampling approach of Russel et al. (2018). For this purpose, the StarBEAST3.*xml* files for each clock model were manually edited to generate a nested-sampling run with a chain length of 10 million, 10 particles, a sub-chain length of 100,000, and a single thread. Analyses were conducted with the Nested Sampling package v1.2.2 in BEAST v2.7.7. Due to time constraints, the marginal likelihood of each clock model was estimated from the combination of 10 independent runs using the same settings. The combined log files were processed with the Nested Sampling Log Analyzer to obtain the MLE.

## ﻿Results

Characteristics of the dataset, including statistical results from the parsimony analysis, are presented in Suppl. material 1: table S3. Recombination testing using RDP v4.101 showed no evidence of recombination events in any of the loci. The estimated Bayesian and ML gene trees are presented in Suppl. material 1: fig. S1, Suppl. material 2, respectively.

### ﻿Strict clock model

The estimated QS values from ASTRAL-III are presented in Suppl. material 3. For the placement of *S. sordida* within the gene trees, the QS metric (q1) was observed as 1.0 under both reference species tree topologies, with the remaining metrics (q2, q3, f1,…, pp3) consistent with this result (see Suppl. material 3 for details). Specifically, under the strict clock phylogeny, the q1 value for the position of *S. sordida* was 0.11 (q2 = 0.41, q3 = 0.48).

Nested sampling analysis showed an MLE of −11652.1489 log units (SD = 1.06) for the assumption of the strict clock model.

Parallel runs of the Bayesian phylogenetic analysis successfully converged on the same parameter space, with effective sample size (ESS) values exceeding 1000 for most parameters. A few parameters related to population sizes (popSize) and tree distances in gene trees exhibited lower ESS values (<200).

**Figure 1.**
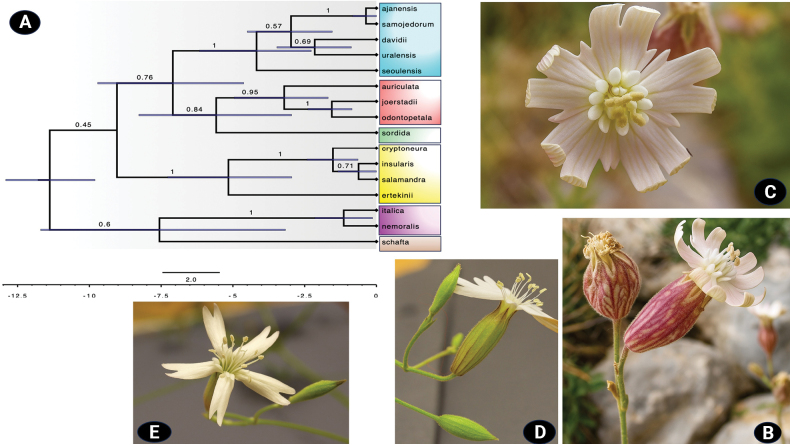
A. Maximum clade credibility species tree estimated from alignments of four nuclear and one chloroplast (cpDNA) region across 16 *Silene* species under a strict molecular clock. This model was preferred according to a model selection procedure based on marginal likelihood estimates of competing models. Phylogenetic analysis was conducted using the multispecies coalescent model in StarBEAST3. Branches are annotated with posterior probabilities, and blue bars at nodes indicate 95% highest posterior density (HPD) intervals for node heights, reflecting uncertainty in divergence times. Tip labels are coloured by sectional assignment within *Silene*. The scale bar represents substitutions per site, and the time axis (in Ma, million years ago) shows estimated divergence times, including the split between S. subg. Behenantha and S. subg. Silene and their respective lineages. Photographs show (B, C) calyx and petal morphology of *S. odontopetala* and (D, E) calyx and petal morphology of *S. sordida* grown under greenhouse conditions. Photo credits: (B, C) Kemal Yıldız; (D, E) Zeynep Toprak. Figures were generated using FigTree v1.4.4 and Inkscape v1.1.1.

The estimated root height of the species tree (mrca.age(SpeciestreerootHeight)) had a mean of 11.38 Ma (95% HPD interval: 9.79–12.95). At the phylogenetic level, the species tree (Fig. 1A) was poorly resolved at deeper nodes, but the main sections were recovered as monophyletic with good support. Members of S. subg. Behenantha clustered together, diverging from members of S. subg. Silene approximately 9.80–12.91 Ma (95% HPD interval). Silene sect. Cryptoneurae was placed as sister to the rest of S. subg. Behenantha, albeit with poor support. Meanwhile, S. sect. Physolychnis was recovered as sister to S. sect. Odontopetalae and *S. sordida* with limited support (PP = 0.76). *S. sordida* and S. sect. Odontopetalae were inferred as sister lineages with moderate support (PP = 0.84), diverging approximately 5.5 Ma (95% HPD interval: 2.95–8.27). Importantly, *S. sordida* showed no direct sister relationship with any specific member of S. sect. Odontopetalae.

Gene tree comparisons revealed that *ITS* and *RPA2* supported a sister relationship between *S. sordida* and S. sect. Odontopetalae. The *rps16* locus also supported this relationship (PP = 0.97). The *RPB2* locus instead strongly supported *S. sordida* as sister to S. sect. Cryptoneurae (PP = 0.99/1.0). The *EST04* locus placed *S. sordida* differently, at a well-supported position (PP = 0.96) with *S. schafta*. None of the loci suggested a direct sister relationship between *S. sordida* and any sampled individuals of *S. odontopetala*.

**Figure 2.**
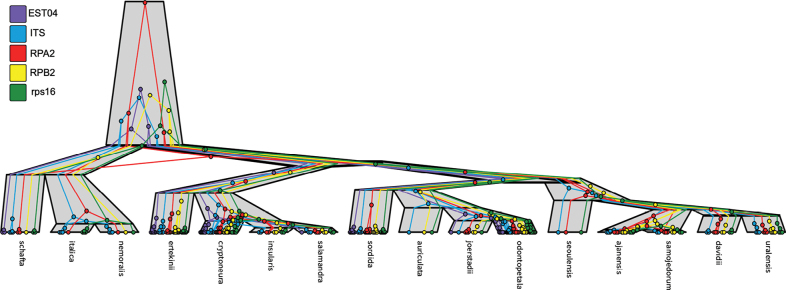
Schematic representation of the compatibility of the five gene trees with their species tree, obtained using UglyTrees (Douglas 2021). Coloured circles represent sampled sequences for 16 species and their inferred coalescent events traced backward in time, as estimated by StarBEAST3 under the multispecies coalescent model. Gray bars delineate species boundaries and the most recent common ancestors of those species. For visualization purposes, the widths of the species branches are not scaled to reflect the effective population sizes of the corresponding lineages.

The overall compatibility of the five gene trees (Suppl. material 1: fig. S1) with the species tree (Fig. 1A) is illustrated in Fig. 2.

### ﻿Relaxed clock model

Nested sampling analysis produced an MLE of −11655.9935 log units with a standard deviation of 0.65 for the relaxed clock model. For the placement of *S. sordida*, the QS value (Suppl. material 3) of q1 was 0.40 (q2 = 0.41, q3 = 0.48).

The mean relative clock rates for each partition against the *RPA2* locus ranged from 0.7001 to 0.8644, showing no significant differences from the scenario where each locus had an independent rate. The mean species coefficient of variation (RateStatLogger.Species.coefficientOfVariation) was below 1.0, with a mean of 0.5217 (95% HPD interval: 0.3257–0.7362) and a standard deviation of 0.109. The estimated root height of the species tree was 11.28 Ma (95% HPD interval: 9.67–12.85).

The species tree (Suppl. material 4) provided well-supported relationships at deeper levels. However, sectional relationships among *Odontopetalae*, *Cryptoneurae*, and Sordidae lacked support. Each section was recovered as monophyletic with high posterior probabilities (PP > 0.90) along the ancestral branches. *S. schafta* was placed apart from other members of S. subg. Silene, diverging early from the rest of S. subg. Behenantha.

In this phylogeny, *S. sordida* was recovered as sister to S. sect. Cryptoneurae, diverging around 5 Ma (95% HPD interval: 3.05–7.57), but this relationship was unsupported (PP = 0.41). Silene sect. Odontopetalae was positioned as sister to the clade containing S. sect. Cryptoneurae and *S. sordida*, but again without support (PP = 0.31).

## ﻿Discussion

### ﻿Species tree, clock model, and time calibration

There were substantial differences among the topologies estimated under both clock models. The phylogeny inferred under the strict clock model (Fig. 1A) aligns with the recent revision by Jafari et al. (2020), which is based on combined *ITS* and *rps16* data from thousands of samples across the entire genus. In contrast, the species tree generated under the relaxed clock model (Suppl. material 4) was highly unresolved because of poor support for the placement of the focal species. Nested sampling analysis provided a statistical basis for comparing the two clock models, yielding MLE scores that favored the strict clock over the relaxed clock by 3.84 log units [RC_MLE (−11655.9935) vs. SC_MLE (−11652.1489)]. Based on the criterion of Kass and Raftery (1995), this difference represents only modest support for the strict clock model, with the standard deviations (SD_SC = 1.06; SD_RC = 0.65) further highlighting the tentative nature of this result. Under the strict clock topology, *S. sordida* and S. sect. Odontopetalae appear as the most likely sister groups (PP = 0.84) among the lineages emphasized in the global revision of the genus by Jafari et al. (2020), though this interpretation remains cautious given the weak statistical foundation and limited support. The estimates of the species tree root age are consistent with previous dating studies of *Silene* (Frajman et al. 2009; Rautenberg et al. 2010; Moiloa et al. 2021), despite the unsatisfactory phylogenetic resolution observed both here and in earlier work. Previous studies (Frajman et al. 2009; Rautenberg et al. 2012) based on the 34–45 Ma caryophyllaceous fossil (Jordan and Macphail 2003) revealed the root age of *Silene* at approximately 11.6 Ma (95% HPD: 9.18–12.17). Using the same calibration data, Moiloa et al. (2021) estimated the root age as 10.7 Ma (95% HPD: 9.02–12.19). The analysis in this study produced a mean root age of 11.3 Ma (95% HPD: 9.79–12.95) under the strict clock model, with slightly lower but comparable estimates under the relaxed clock model.

Both strict and relaxed clock models can yield robust divergence time estimates, particularly when evolutionary rates are relatively homogeneous (Ho et al. 2005). However, although the sampling included representatives from the two main subgenera, the number of analysed samples was rather limited given the vast diversity within *Silene*, which may have affected estimated rates. There is substantial diversity in rate evolution models and their implementations, and previous studies have shown that these can influence divergence time estimates as much as calibration information (Mello and Schrago 2024). While multiple relaxed clock models may offer a more biologically realistic approach to modelling rate variation among lineages, their use must be balanced against the risk of overparameterization, which can pose a significant challenge when analysing uninformative datasets (Ho and Lanfear 2010). In relaxed clock models, additional parameters such as the coefficient of rate variation and covariance between branch rates help assess model fit, though empirical evidence for autocorrelated rates remains weak (Drummond et al. 2006). In this study, the mean coefficient of variation of species tree branch rates was 0.5, lower than 1.0 (values > 1.0 indicate increasing rate heterogeneity among branches; Drummond et al. 2006). Additionally, the strict clock model yielded narrower HPD interval widths (3.16), consistent with findings by Ho et al. (2005), suggesting reduced uncertainty compared with the relaxed clock model (3.18), although the difference is likely not significant in this case. All these factors may help explain the higher PP values observed in the upper branches of the species tree estimated under the strict clock model (Fig. 1A).

The many evolutionary questions requiring a time-calibrated phylogeny highlight the need for precise and accurate divergence time methods. Bayesian and full-likelihood methods (e.g., those based on the MSC) have become prominent tools for estimating divergence times. Although computationally intensive, these methods account for uncertainty in gene tree topologies and coalescent times and have improved with advancements in MCMC proposal algorithms (Rannala and Yang 2003; Ogilvie et al. 2017; Rannala and Yang 2017; Douglas et al. 2022). The StarBEAST3 method used here is an efficient Bayesian inference approach well suited to dating analyses, as it integrates information from gene trees while accommodating their uncertainties, along with several technical improvements (Douglas et al. 2022). Despite certain limitations, StarBEAST3 analysis based on five loci estimated the divergence of *S. sordida* and S. sect. Odontopetalae at approximately 5.5 Ma (95% HPD: 2.9–8.9), placing it at the end of the Miocene (ca. 5.3–23 Ma), shortly after the diversification of ancestral *Silene* lineages and some other angiosperm groups originating in the Mediterranean Basin (Thompson 2005). However, it should be noted that speciation events are often more recent than gene divergences because of coalescent processes (Gillespie and Langley 1979), suggesting that the genetic divergence of *S. sordida* from the ancestral *Odontopetalae* lineage may have occurred even earlier, while acknowledging the limited support for this relationship. The accuracy of StarBEAST3, as with most dating inference methods, may be affected by the number and placement of node calibrations that help prevent calibration error from propagating across the tree. Unfortunately, in the absence of fossil, biogeographic, or molecular rate evidence, the calibration for this study relied solely on previously published estimates of *Silene*’s root age (Frajman et al. 2009; Rautenberg et al. 2010; Moiloa et al. 2021). The limited availability of calibration data remains a key constraint in dating studies of *Silene*; therefore, future research should aim to incorporate multiple node calibrations to improve the accuracy and robustness of divergence time estimates in the genus.

### ﻿Phylogenetic relationships of S. odontopetala and S. sordida

Determining phylogenetic relationships within *Silene* requires an understanding of the evolutionary events that have shaped current patterns. In line with previous findings (Jafari et al. 2020; Toprak and Yıldız 2022), the results confirm a sister relationship between sections *Odontopetalae* and *Sordidae*, although the anticipated strong statistical support was lacking. Compared with earlier studies (Jafari et al. 2020) using species tree approaches, increasing the sampling size of the focal taxa substantially improved the support (PP = 0.84) for the ancestral branch diverging into both sections, although the evidence is still limited.

On the other hand, the limited support found in the results could be due to several factors, such as the evolutionary history of individual genes (Suppl. material 1: fig. S1). For instance, the *RPB2* locus Suppl. material 1: fig. S1) strongly supports (PP = 0.99) a sister relationship between sections *Cryptoneurae* and *Sordidae*, which contrasts with the relationships suggested by the other four loci. Additionally, the *EST04* (Suppl. material 1: fig. S1) locus produced conflicting results, rejecting a close relationship between *S. sordida* and either *Cryptoneurae* or *Odontopetalae*. Notably, analyses of this locus lacked any representatives from S. sect. Physolychnis. Another potential source of weak support could be the limited representation of genetic diversity in the less-emphasized sections, which include the largest groups within the genus. Silene sect. Physolychnis s.l., with approximately 162 species, is the largest section in the genus, followed by S. sect. Siphonomorpha and S. sect. Auriculatae, according to the recently revised infrageneric taxonomy of *Silene*. Although broader taxonomic inclusion was not feasible due to various constraints, this study still incorporated all major lineages potentially sharing close ties to *S. sordida*, as inferred from the most comprehensive recent revision of the genus (Jafari et al. 2020).

Despite *S. odontopetala* being relatively well represented in the study, the phylogenetic relationships and boundaries of S. sect. Odontopetalae remain largely unclear. Traditional classifications based on morphological traits such as carpel number are challenged by molecular studies, which suggest that three-carpelled species like *S. odontopetala* are more closely related to S. sect. Physolychnis. This supports a broader, monophyletic *Physolychnis* that includes both three- and five-carpelled species across Eurasia and North America (Petri and Oxelman 2011). These findings point to the likely paraphyly of the *Odontopetalae* group and the need for a taxonomic revision.

The observed gene tree incongruence—a common issue in phylogenetics—among the analysed loci can result from biological phenomena such as incomplete lineage sorting (ILS), hybridization, recombination, and paralogy, among others (Degnan and Rosenberg 2006, 2009). To understand the source of incongruence between the gene trees and the estimated species tree, recombination was first ruled out, as no signal was detected by the recombination detection program RDP v4.101. Paralogy could potentially explain the sister relationship between *S. sordida* and sect. Cryptoneurae inferred by the *RPB2* locus (Suppl. material 1: fig. S1). However, this scenario necessitates multiple ad hoc assumptions. For example, it would require at least one duplication event in the ancestral lineage that diverged into sections *Cryptoneurae*, *Odontopetalae*, and *S. sordida*, followed by the loss of a duplicate in *Odontopetalae*. A notable feature of this study is the inclusion of multiple individuals per focal species. In the *RPB2* gene phylogenies (both Bayesian and ML), samples of *S. sordida* and sect. Cryptoneurae do not intermingle. If paralogy were present, individuals carrying the same paralogous copy would be expected to cluster together, forming distinct clades separate from those with the alternative copy; however, no such pattern was observed. While it is theoretically possible that all analysed individuals from both groups possess the same *RPB2* paralog, this scenario would be highly unparsimonious and less plausible than alternative explanations. Nevertheless, duplication events of the RNAP gene family have been reported in *Silene* (Popp and Oxelman 2004; Petri and Oxelman 2011; Petri et al. 2013). A more plausible explanation involves ILS. This occurs when the coalescence of alleles predates the speciation events of the taxa they represent, potentially explaining the more recent coalescence of *S. sordida* and *Cryptoneurae* alleles in the *RPB2* locus than those of *Odontopetalae*. Importantly, this phenomenon is well accounted for in phylogenetic analyses employing methods based on the MSC model, such as StarBEAST3 used here. ILS is typically more likely when speciation events occur in rapid succession. Given the relatively long divergence interval of ~5.5 Ma between *S. sordida* and the sections *Odontopetalae* and *Cryptoneurae* (as suggested by species trees under both clock models, though weakly supported), the probability of ILS seems low, though it cannot be entirely excluded. This interpretation is complicated by the quartet support (QS) results from ASTRAL-III. Branches with high q1 values (≥0.8) are generally considered well supported and congruent across gene trees, whereas low q1 values, or q1 values similar to q2 or q3, indicate strong gene tree discordance and phylogenetic uncertainty, often arising from rapid speciation or ILS. In the strict clock reference tree, the q1 value for *S. sordida* is observed as only 0.11, signaling substantial discordance. Under the relaxed clock topology, q1 and q3 for the same position are 0.40 and 0.48, respectively. By contrast, the ML gene trees show a q1 value of 1.0 for *S. sordida* when compared against both reference tree topologies (Suppl. material 3). QS values are useful (Pease et al. 2018) for assessing the reliability of phylogenetic certainty; however, they are also prone to errors associated with the user-provided reference tree, in addition to sampling effects, the local-search algorithm, and other factors (Sayyari et al. 2018).

**Figure 3.**
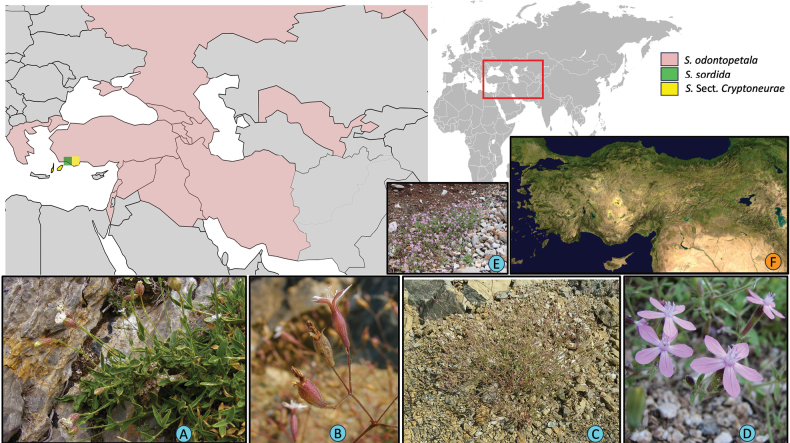
Map illustrating the approximate known geographic distributions of *Silene sordida* (green), *S. odontopetala* (rose), and sect. Cryptoneurae (yellow). Distribution data were compiled from GBIF (gbif.org), “Flora of Turkey and the East Aegean Islands” (Davis 1965–1985), and POWO (powo.science.kew.org) based on country-level records. Photographs: A. *S. odontopetala* (habit); B. *S. sordida* (flower); C. *S. sordida* (habit); D. *S. ertekinii* (flower); E. *S. ertekinii* (habit); F. geographical map of Anatolia. Photo credits: A–C. Kemal Yıldız; D–E. Zeynep Toprak. Figure generated using Python 3.13, Inkscape 1.1.1, and Microsoft PowerPoint 16.78.

Hybridization, on the other hand, is a well-known process in *Silene*. Both ancient and recent hybridization events play a key role in shaping phylogenetic relationships and understanding the evolutionary history of the genus (Erixon and Oxelman 2008; Rautenberg et al. 2010; Petri et al. 2013; Pfeil et al. 2017). Similarly, *S. sordida* was thought to represent a potential homoploid hybrid (Schumer et al. 2014) in an earlier study by Erixon and Oxelman (2008). On the other hand, ILS and hybridization may occur simultaneously (Seehausen 2004), and it can be difficult to distinguish between them without a statistical framework (Holland et al. 2008). However, a necessary condition for hybridization is that it must occur after the divergence of the lineages; based on this and the observed pattern, recent hybridization seems unlikely since *S. sordida* shows no close relationship to any existing members of S. sect. Cryptoneurae, regardless of their geographic proximity (Fig. 3). An ancient hybridization with the ancestral lineage of S. sect. Cryptoneurae might have occurred. However, the timing from the species tree (Fig. 1A) shows scant evidence for this, as the divergence of *S. sordida* from the ancestral lineage of S. sect. Odontopetalae (5.58 Ma) largely overlaps with the diversification of species constituting S. sect. Cryptoneurae (5.15 Ma). Although the divergence of *S. sordida* is slightly earlier, the upper and lower bounds of the nodes forming *S. sordida* (2.95–8.27 Ma) and the diversification of species of sect. Cryptoneurae (2.95–7.27 Ma) render this possibility questionable. Notably, studies have revealed introgression between species from different subgenera of *Silene* approximately 6.6 Ma (Petri et al. 2013). Overall, while several hypotheses remain possible, the limited evidence points most plausibly—though not decisively—toward ILS.

### ﻿Biogeographic implications and convergent morphological evolution in *S. sordida*

The use of molecular markers has helped clarify the evolutionary relationships between *S. sordida* and members of the *Odontopetalae* group, offering insights into the biogeographic history of *S. sordida*. Despite their close genetic relationship, *S. sordida* is morphologically and ecologically distinct from *S. odontopetala*.

*Silene odontopetala* is characterized by linear-lanceolate to rectangular to petiolate basal leaves, linear-lanceolate to obovate cauline leaves, a broadly tubular and prominently inflated fruiting calyx, winged petal claws, and deeply lobed petal limbs (Fig. 1B, C). It also has a unique seed morphology, with smooth, irregularly triangular seeds featuring dense capitate tubercles and a recessed hilum (Chowdhuri 1957; Gholipour and Maroofi 2011; Toprak and Yıldız 2022). However, correlations among these morphological traits in *S. odontopetala* are reported to be weak (Coode and Cullen 1967).

Ecologically, *S. odontopetala* is a perennial species adapted to rocky, calcareous cliffs at high elevations (500–4000 m) across the eastern Mediterranean to Central Asia (Gholipour and Maroofi 2011). In contrast, *S. sordida* is an annual species restricted to serpentine slopes in lower-altitude areas of southwestern Anatolia. Even though *S. sordida* is genetically similar to the *S. odontopetalae* group, it displays morphological characteristics such as an annual habit, globular seeds, slightly dissected pink petal limbs, lax dichasial inflorescences, and glandular hairs, features similar to those of S. sect. Cryptoneurae (Aydin et al. 2014a; Toprak and Yıldız 2022). This pattern suggests that the similarity results from convergent evolution, a phenomenon well known in *Silene* (Naciri et al. 2022). Confirmed by the present results, homoplasy in diagnostic morphological traits poses a significant challenge in *Silene* taxonomy. Features such as style number, capsule teeth, life form, calyx inflation, indumentum, and venation have evolved independently in different lineages, complicating phylogenetic interpretation (Oxelman and Lidén 1995; Oxelman et al. 2001; Popp et al. 2005; Petri and Oxelman 2011; Aydin et al. 2014a; Frajman et al. 2018). For instance, calyx inflation—once considered taxonomically informative—has evolved independently in several genera now classified within *Silene* (e.g., *Cucubalus* L., *Melandrium* Röhl., *Schischkiniella* Steenis) and does not reflect monophyletic groupings (Eggens 2006; Rautenberg et al. 2012).

Biogeographically, *Silene* is believed to have originated in the Mediterranean Basin during the mid-late Miocene, a region recognized as a biodiversity hotspot due to its complex tectonic and climatic history (Meulenkamp and Sissingh 2003; Thompson 2005; Blondel et al. 2010). Early diversification in *Silene*, as in many angiosperms, was likely driven by geological events such as the Messinian salinity crisis (~5.96–5.33 Ma), which temporarily exposed land bridges and enabled dispersal followed by isolation (Penzo et al. 1998; Krijgsman 2002; Sanmartín 2003; Tsigenopoulos et al. 2003; Carlson et al. 2012; Özüdoğru et al. 2015; Costa et al. 2022).

Although the distributions of *S. sordida* and *S. odontopetala* overlap in southwestern Anatolia (Fig. 3), their distinct ecological preferences suggest that ecological speciation is more plausible than allopatric speciation. In Anatolia, *S. odontopetala* is usually found at elevations above 1000 m, whereas *S. sordida* is adapted to low-altitude serpentine soils. These contrasting ecological preferences, coupled with a lack of clear evidence for recombination or hybridization, point to intrinsic reproductive isolation driven by ecological differentiation (Coyne and Orr 2004; Nosil et al. 2008; Muir et al. 2012; Seehausen et al. 2014; Guirao-Rico et al. 2017). The ecological divergence of *S. sordida* and the *S. odontopetalae* group may have been shaped by contrasting environmental pressures. For instance, the presence of *S. odontopetala* in the Hyrcanian forests of northern Iran is linked to its status as an ecological indicator of this ancient biome (Naqinezhad and Esmailpoor 2017), while *S. sordida* is a serpentine specialist, similar to *S. cryptoneura* Stapf and *S. ertekinii* Aydın & Oxelman, which occupy nearby scree slopes and undisturbed roadsides (Fig. 3B–E). Other S. sect. Cryptoneurae species, including *S. salamandra* Pampanini and *S. insularis* Barbey, are island endemics to Rhodes and Karpathos, respectively. The results suggest that the divergence between mainland *S. cryptoneura* and its insular relatives occurred around 1.5 Ma, likely driven by tectonic uplift and submergence events ca. 3–4 Ma near Rhodes Island (NASA Earth Observatory, available at earthobservatory.nasa).

An alternative scenario attributes lineage divergence to the Messinian salinity crisis (~5.3 Ma; Garcia-Castellanos and Villaseñor 2011), when the desiccation and refilling of the Mediterranean reshaped habitats. Sea-level low stands may have supported broader ancestral ranges of *S. sordida*, later fragmented by rising seas, with populations persisting in serpentine refugia (*S. sordida*) or shifting to high-altitude habitats (*S. odontopetala*). The uplift of the Anatolian Plateau in the mid–late Miocene (Şaroğlu and Yılmaz 1986) further expanded montane habitats, likely enabling eastward spread of *S. odontopetala* into Central Asia. Subsequent Pleistocene glacial–interglacial cycles (~2.5 Ma onwards) reinforced isolation and divergence (Davis 1965; Sanmartín 2003; Ansell et al. 2011).

In summary, the divergence of *S. sordida* from the *S. odontopetalae* lineage likely involved a complex interplay of ecological specialization, historical vicariance, and convergent morphological evolution. While molecular data suggest a close relationship, *S. sordida*’s distinct morphology and ecological adaptations point to an independent evolutionary trajectory. These findings reflect broader diversification patterns within *Silene*, shaped by the dynamic geological and climatic history of the eastern Mediterranean and Anatolia.

## ﻿Additional information

### Use of AI

No use of AI was reported.

### Author ORCIDs

Zeynep Toprak https://orcid.org/0000-0002-2103-4141

### Data availability

The GenBank accession codes of the DNA sequences and locality information of the plant samples are provided in Suppl. material 1: table S1.

## Supplementary materials

Supplementary material 1 Supplementary information Data type docx Explanation note **table S1.** List of analysed specimens. **table S2.** Details of primer sequences and applied PCR programs. **table S3.** Statistics of the parsimony analysis for the used loci. **figure S1.** Bayesian gene trees inferred from five loci. https://binary.pensoft.net/file/1448139This dataset is made available under the Open Database License (http://opendatacommons.org/licenses/odbl/1.0/). The Open Database License (ODbL) is a license agreement intended to allow users to freely share, modify, and use this Dataset while maintaining this same freedom for others, provided that the original source and author(s) are credited.Author: Zeynep Toprak

Supplementary material 2 Maximum likelihood gene trees inferred from five loci Data type txt https://binary.pensoft.net/file/1448140This dataset is made available under the Open Database License (http://opendatacommons.org/licenses/odbl/1.0/). The Open Database License (ODbL) is a license agreement intended to allow users to freely share, modify, and use this Dataset while maintaining this same freedom for others, provided that the original source and author(s) are credited.Author: Zeynep Toprak

Supplementary material 3 Quartet sampling (QS) values obtained from ASTRAL-III Data type docx https://binary.pensoft.net/file/1448141This dataset is made available under the Open Database License (http://opendatacommons.org/licenses/odbl/1.0/). The Open Database License (ODbL) is a license agreement intended to allow users to freely share, modify, and use this Dataset while maintaining this same freedom for others, provided that the original source and author(s) are credited.Author: Zeynep Toprak

Supplementary material 4 Species phylogeny inferred under a relaxed clock model Data type png https://binary.pensoft.net/file/1448142This dataset is made available under the Open Database License (http://opendatacommons.org/licenses/odbl/1.0/). The Open Database License (ODbL) is a license agreement intended to allow users to freely share, modify, and use this Dataset while maintaining this same freedom for others, provided that the original source and author(s) are credited.Author: Zeynep Toprak

Supplementary material 5 Species tree estimated under a strict clock model Data type tre https://binary.pensoft.net/file/1448143This dataset is made available under the Open Database License (http://opendatacommons.org/licenses/odbl/1.0/). The Open Database License (ODbL) is a license agreement intended to allow users to freely share, modify, and use this Dataset while maintaining this same freedom for others, provided that the original source and author(s) are credited.Author: Zeynep Toprak
