# Supplementary material for: ﻿Phylogenetic analysis suggests early divergence followed by convergent morphological evolution in the Silene sections Odontopetalae and Sordidae (Caryophyllaceae)
Source: PhytoKeys. 2025 Oct 24;265:123–45. doi: 10.3897/phytokeys.265.165998 (PMC12579334; doi:10.3897/phytokeys.265.165998)
Supplement: Supplementary material 1 — Supplementary information [file phytokeys-265-123_article-165998__-s001.docx]

**SUPPLEMENTARY MATERIAL**

**Table S1**. List of the analysed Specimens.

| **Specimen** | **Locus** | **Gene Bank- ID** | **Author & Specimen Voucher** | **Location** |
| --- | --- | --- | --- | --- |
| S_cryptoneura_14858_5847 | EST04 | PX262413 | Bengt Oxelman 2513 GB | Turkey |
| S_cryptoneura_14849_5848 | EST04 | PX262414 | Bengt Oxelman 2504 GB | Turkey |
| S_cryptoneura_14858_6048 | EST04 | PX262415 | Bengt Oxelman 2513 GB | Turkey |
| S_cryptoneura_1115_6194 | EST04 | PX262416 | Bengt Oxelman 1628 GB | Turkey: Antalya |
| S_ertekinii_15351_5820 | EST04 | PX262417 | Zeynep Aydın 31 GB | Turkey: Antalya |
| S_ertekinii_15352_5821 | EST04 | PX262418 | Zeynep Aydın 28 GB | Turkey: Antalya |
| S_ertekinii_15178_5825 | EST04 | PX262419 | Zeynep Aydın 36 GB | Turkey: Antalya |
| S_ertekinii_15178_6021 | EST04 | PX262420 | Zeynep Aydın 36 GB | Turkey: Antalya |
| S_joerstadii_7316_6058 | EST04 | PX262422 | Hedge and Wendelbo W 8992 GB | Afghanistan: Paktiva |
| S_salamandra_15345_5849 | EST04 | PX262421 | Bengt Oxelman 2541 GB | Greece |
| S_schafta_2474_6265 | EST04 | PX262411 | Bengt Oxelman 2264 GB | Cultivated Plants (Iran?) |
| S_schafta_7615_6266 | EST04 | PX262412 | Magnus Popp 1053 GB | Cultivated Plants (UPS) |
| S_sordida_14840_5845 | EST04 | PX262408 | Bengt Oxelman 2495 GB | Turkey: Mughla |
| S_sordida_14838_5846 | EST04 | PX262409 | Bengt Oxelman 2493 GB | Turkey: Mughla |
| S_sordida_14835_6053 | EST04 | PX262410 | Bengt Oxelman 2492 GB | Turkey: Mughla |
| S_cryptoneura_30526 | EST04 | PX262405 | K. Yıldız 0318 | Turkey: Antalya |
| S_cryptoneura_30527 | EST04 | PX262406 | K. Yıldız 0397-1 | Turkey: Antalya |
| S_cryptoneura_30528 | EST04 | PX262407 | K. Yıldız 0551 | Turkey: Antalya |
| S_ertekinii_30525 | EST04 | PX262404 | K. Yıldız 0558 | Turkey: Antalya |
| S_odontopetala_30518 | EST04 | PX262397 | K. Yıldız 013-2 | Turkey: Kahramanmaraş |
| S_odontopetala_30519 | EST04 | PX262398 | K. Yıldız 0299-4 | Turkey: Isparta |
| S_odontopetala_30520 | EST04 | PX262399 | K. Yıldız 0593-2 | Turkey: Uşak |
| S_odontopetala_30521 | EST04 | PX262400 | K. Yıldız 337-2 | Turkey: Bayburt |
| S_odontopetala_30522 | EST04 | PX262401 | K. Yıldız 0369-4 | Turkey: Isparta |
| S_sordida_30523 | EST04 | PX262402 | K. Yıldız 0392 | Turkey: Mugla |
| S_sordida_30524 | EST04 | PX262403 | K. Yıldız 0295-3 | Turkey: Mugla |
| S_ajanensis_6362_788 | ITS | AJ831780 | Magnus Popp 200206 UPS | Russia |
| S_ajanensis_7049_2040 | ITS | KX757376 | Anja Rautenberg 68 UPS | Russia: Magadan |
| S_ajanensis_12534_2246 | ITS | KX757384 | Khokhrjakov LE | Russia |
| S_auriculata_14154_3315 | ITS | KX757292 | Baden and Franzén 795 Strid | Greece: Korinthos |
| S_cryptoneura_1115_4896 | ITS | KX757319 | Bengt Oxelman 1628 GB | Turkey: Antalya |
| S_cryptoneura_14849_6705 | ITS | KX757321 | Bengt Oxelman 2504 GB | Turkey |
| S_cryptoneura_14879_6707 | ITS | KX757323 | Bengt Oxelman 2524 GB | Turkey |
| S_cryptoneura_1140_6710 | ITS | KX757320 | Bengt Oxelman 1653 GB | Turkey: Antalya |
| S_cryptoneura_17888_6711 | ITS | KX757322 | Deniz 1573 GB | Turkey |
| S_davidii_13374_2598 | ITS | FN821093 | Frida Eggens 85 GB | China: Sichuan |
| S_davidii_13375_2600 | ITS | KX757367 | Frida Eggens 86 GB | China: Sichuan |
| S_ertekinii_1177_1863 | ITS | X86823 | Bengt Oxelman 1691 GB | Turkey: Antalya |
| S_ertekinii_15351_6703 | ITS | KX757328 | Zeynep Aydın 31 GB | Turkey: Antalya |
| S_ertekinii_15178_6704 | ITS | KX757330 | Zeynep Aydın 36 GB | Turkey: Antalya |
| S_ertekinii_15184_6708 | ITS | KX757329 | Zeynep Aydın 38 GB | Turkey: Antalya |
| S_ertekinii_15153_6709 | ITS | KX757327 | Zeynep Aydın 31 GB | Turkey: Antalya |
| S_insularis_12262_1851 | ITS | X86822 | Raus 9700 UPS | Greece: Dodekanisou |
| S_insularis_15372_6706 | ITS | KX757324 | Raus 9700 UPS | Greece: Dodekanisou |
| S_italica_12804_1924 | ITS | U30960U30986 |  | ? |
| S_italica_15252_6320 | ITS | PX218332 | Zeynep Aydın 49 GB | Turkey |
| S_italica_17755_6362 | ITS | KX757559 | Bengt Oxelman 2619 GB | Turkey |
| S_italica_18998_7149 | ITS | LC424095 | Eslami TUH | Iran: Mazandaran |
| S_joerstadii_7316_957 | ITS | KX757293 | Hedge and Wendelbo W 8992 GB | Afghanistan: Paktiva |
| S_nemoralis_2465_1542 | ITS | KX757485 | Bengt Oxelman 2259 GB | Austria |
| S_nemoralis_2465_1846 | ITS | X86863 | Bengt Oxelman 2259 GB | Austria |
| S_odontopetala_7591_1837 | ITS | X86833 | HOLUBEC -94 | Turkey |
| S_odontopetala_14169_3312 | ITS | KX757294 | Görk et al. 23817 Strid | Turkey |
| S_odontopetala_15381_6354 | ITS | PX218331 | Bengt Oxelman 2612 GB | Iran |
| S_odontopetala_19032_7108 | ITS | LC424054 | A. Gholipour SPNH | Iran: Tehran |
| S_odontopetala_19033_7109 | ITS | LC424055 | A. Gholipour SPNH | Iran: Mazandaran |
| S_salamandra_2410_14 | ITS | KX757325 | Bengt Oxelman 2205 GB | Greece: Dodekanisou |
| S_salamandra_2410_4891 | ITS | KX757326 | Bengt Oxelman 2205 GB | Greece: Dodekanisou |
| S_samojedorum_12338_2027 | ITS | KX757374 | H. Solstad, R. Elven SUP-04-3871 O | Russia: Sakha Republic (Yakutia) |
| S_samojedorum_12339_2034 | ITS | KX757380 | Peter Schönswetter, Andreas Tribsch T601 GB | Russia |
| S_samojedorum_12396_2247 | ITS | KX757375 | V. V. Petrovskij LE | Russia |
| S_schafta_7615_1788 | ITS | AJ831792 | Magnus Popp 1053 GB | Cultivated Plants |
| S_schafta_2474_1827 | ITS | X86852 | Bengt Oxelman 2264 GB | Cultivated Plants (İran?) |
| S_schafta_19029_7132 | ITS | LC424078 | SPNH | Iran: Mazandaran |
| S_seoulensis_7812_1971 | ITS | KX757373 | Hong and Han 13420001 UPS | Korea |
| S_sordida_2411_967 | ITS | X86824 | Bengt Oxelman 2206 GB | Turkey: Mughla |
| S_uralensis_7614_42 | ITS | KX757348 | Magnus Popp 1052 UPS | Cultivated Plants |
| S_uralensis_6364_791 | ITS | KX757341 | Fredskild and Dalgaard 87837 C | Greenland |
| S_uralensis_7023_838 | ITS | KX757342 | Inger Skrede SUP02-38-8 O | Greenland |
| S_uralensis_7587_841 | ITS | AJ831781 | Reidar Elven et al. SUP02-1002-2 O | Alaska |
| S_uralensis_6432_1002 | ITS | KX757346 | Lidén 301 UPS | Mongolia |
| S_uralensis_6432_1794 | ITS | AJ831785 | Lidén 301 UPS | Mongolia |
| S_uralensis_7023_1795 | ITS | AJ831784 | Inger Skrede SUP02-38-8 O | Greenland |
| S_uralensis_6364_1796 | ITS | AJ831783 | Fredskild and Dalgaard 87837 C | Greenland |
| S_uralensis_14348_3642 | ITS | KX757345 | Brysting AK-4645-4 | Canada |
| S_uralensis_7053_3646 | ITS | KX757347 | Anja Rautenberg 72 UPS | Norway |
| S_uralensis_7029_3647 | ITS | KX757344 | Anja Rautenberg 65 UPS | Alaska |
| S_nemoralis_24047_8803 | ITS | KX449849 | Monserrat 104476 G | Spain: Huesca |
| S_nemoralis_24051_9710 | ITS | KX449850 | Jeanmonod, D. - G | Italy: Piemont |
| S_nemoralis_24048_9717 | ITS | KX449861 | Coste s.n. G | France: Aveyron |
| S_nemoralis_24062_9724 | ITS | KX449892 | Jeanmonod, D. & Naciri, Y. 7915 G | Austria: Styria |
| S_nemoralis_24068_9731 | ITS | KX449899 | Jeanmonod, D. & Naciri, Y. 7920 G | Slovakia: Banská Bystrica |
| S_cryptoneura_30526 | ITS | PX218328 | K. Yıldız 0318 | Turkey: Antalya |
| S_cryptoneura_30527 | ITS | PX218329 | K. Yıldız 0397-1 | Turkey: Antalya |
| S_cryptoneura_30528 | ITS | PX218330 | K. Yıldız 0551 | Turkey: Antalya |
| S_ertekinii_30525 | ITS | PX218327 | K. Yıldız 0558 | Turkey: Antalya |
| S_odontopetala_30518 | ITS | PX218320 | K. Yıldız 013-2 | Turkey: Kahramanmaraş |
| S_odontopetala_30519 | ITS | PX218321 | K. Yıldız 0299-4 | Turkey: Isparta |
| S_odontopetala_30520 | ITS | PX218322 | K. Yıldız 0593-2 | Turkey: Uşak |
| S_odontopetala_30521 | ITS | PX218323 | K. Yıldız 337-2 | Turkey: Bayburt |
| S_odontopetala_30522 | ITS | PX218324 | K. Yıldız 0369-4 | Turkey: Isparta |
| S_sordida_30523 | ITS | PX218325 | K. Yıldız 0392 | Turkey: Mugla |
| S_sordida_30524 | ITS | PX218326 | K. Yıldız 0295-3 | Turkey: Mugla |
| S_ajanensis_7441_641 | RPA2 | AJ629297 | Mikhajlova [? | Russia: Yakutia |
| S_ajanensis_6362_771 | RPA2 | AJ634165 | Magnus Popp 200206 UPS | Russia |
| S_ajanensis_6362_818 | RPA2 | AJ634166 | Magnus Popp 200206 UPS | Russia |
| S_ajanensis_6362_857 | RPA2 | AJ634167 | Magnus Popp 200206 UPS | Russia |
| S_ajanensis_7441_1294 | RPA2 | AJ634163 | Mikhajlova [? | Russia: Yakutia |
| S_ajanensis_7441_1295 | RPA2 | AJ634164 | Mikhajlova [? | Russia: Yakutia |
| S_ajanensis_12534_2121 | RPA2 | KC522759 | Khokhrjakov LE | Russia |
| S_ajanensis_7049_2139 | RPA2 | KC522763 | Anja Rautenberg 68 UPS | Russia: Magadan |
| S_ajanensis_12534_2142 | RPA2 | KC522764 | Khokhrjakov LE | Russia |
| S_cryptoneura_1115_2367 | RPA2 | KC522775 | Bengt Oxelman 1628 GB | Turkey: Antalya |
| S_cryptoneura_14858_5311 | RPA2 | PX148660 | Bengt Oxelman 2513 GB | Turkey |
| S_cryptoneura_14849_5312 | RPA2 | PX148661 | Bengt Oxelman 2504 GB | Turkey |
| S_cryptoneura_14849_5313 | RPA2 | PX148662 | Bengt Oxelman 2504 GB | Turkey |
| S_davidii_13375_2593 | RPA2 | KC522792 | Frida Eggens 86 GB | China: Sichuan |
| S_davidii_13374_2634 | RPA2 | HM595179 | Frida Eggens 85 GB | China: Sichuan |
| S_davidii_13374_2635 | RPA2 | HM595178 | Frida Eggens 85 GB | China: Sichuan |
| S_ertekinii_15178_4901 | RPA2 | PX148663 | Zeynep Aydın 36 GB | Turkey: Antalya |
| S_ertekinii_15352_5011 | RPA2 | PX148666 | Zeynep Aydın 28 GB | Turkey: Antalya |
| S_ertekinii_15351_5484 | RPA2 | PX148667 | Zeynep Aydın 31 GB | Turkey: Antalya |
| S_ertekinii_15351_5485 | RPA2 | PX148668 | Zeynep Aydın 31 GB | Turkey: Antalya |
| S_insularis_15372_5578 | RPA2 | PX148672 | Raus 9700 UPS | Greece: Dodekanisou |
| S_insularis_15372_5579 | RPA2 | PX148673 | Raus 9700 UPS | Greece: Dodekanisou |
| S_italica_15252_6245 | RPA2 | PX148665 | Zeynep Aydın 49 GB | Turkey |
| S_joerstadii_7316_5582 | RPA2 | PX148674 | Hedge and Wendelbo W 8992 GB | Afghanistan: Paktiva |
| S_joerstadii_7316_5588 | RPA2 | PX148675 | Hedge and Wendelbo W 8992 GB | Afghanistan: Paktiva |
| S_joerstadii_7316_5589 | RPA2 | PX148664 | Hedge and Wendelbo W 8992 GB | Afghanistan: Paktiva |
| S_nemoralis_2465_1551 | RPA2 | KC522743 | Bengt Oxelman 2259 GB | Austria |
| S_salamandra_2410_4894 | RPA2 | PX148671 | Bengt Oxelman 2205 GB | Greece: Dodekanisou |
| S_salamandra_15345_5576 | RPA2 | PX148669 | Bengt Oxelman 2541 GB | Greece |
| S_salamandra_15345_5577 | RPA2 | PX148670 | Bengt Oxelman 2541 GB | Greece |
| S_samojedorum_12339_2029 | RPA2 | KC522755 | Peter Schönswetter, Andreas Tribsch T601 GB | Russia |
| S_samojedorum_12338_2131 | RPA2 | KC522761 | H. Solstad, R. Elven SUP-04-3871 O | Russia: Sakha Republic (Yakutia) |
| S_samojedorum_12338_2132 | RPA2 | KC522762 | H. Solstad, R. Elven SUP-04-3871 O | Russia: Sakha Republic (Yakutia) |
| S_schafta_7615_587 | RPA2 | AJ629305 | Magnus Popp 1053 GB | Cultivated Plants |
| S_schafta_2474_4881 | RPA2 | KC522812 | Bengt Oxelman 2264 GB | Cultivated Plants (Iran?) |
| S_seoulensis_7812_1986 | RPA2 | KC522748 | Hong and Han 13420001 UPS | Korea |
| S_sordida_7824_2515 | RPA2 | KC522787 | Bengt Oxelman 2206 GB | Turkey: Mughla |
| S_sordida_14838_5309 | RPA2 | PX148658 | Bengt Oxelman 2493 GB | Turkey: Mughla |
| S_sordida_14838_5310 | RPA2 | PX148659 | Bengt Oxelman 2493 GB | Turkey: Mughla |
| S_sordida_14840_5427 | RPA2 | PX148656 | Bengt Oxelman 2495 GB | Turkey: Mughla |
| S_sordida_14840_5428 | RPA2 | PX148657 | Bengt Oxelman 2495 GB | Turkey: Mughla |
| S_uralensis_7023_894 | RPA2 | PX148654 | Inger Skrede SUP02-38-8 O | Greenland |
| S_uralensis_7587_896 | RPA2 | AJ634179 | Reidar Elven et al. SUP02-1002-2 O | Alaska |
| S_uralensis_6432_1006 | RPA2 | PX148655 | Lidén 301 UPS | Mongolia |
| S_uralensis_14348_4726 | RPA2 | KC522806 | Brysting AK-4645-4 | Canada |
| S_cryptoneura_30527 | RPA2 | PX148647 | K. Yıldız 0397-1 | Turkey: Antalya |
| S_cryptoneura_30528 | RPA2 | PX148646 | K. Yıldız 0551 | Turkey: Antalya |
| S_odontopetala_30519 | RPA2 | PX148650 | K. Yıldız 0299-4 | Turkey: Isparta |
| S_odontopetala_30520 | RPA2 | PX148651 | K. Yıldız 0593-2 | Turkey: Uşak |
| S_odontopetala_30521 | RPA2 | PX148652 | K. Yıldız 337-2 | Turkey: Bayburt |
| S_odontopetala_30522 | RPA2 | PX148653 | K. Yıldız 0369-4 | Turkey: Isparta |
| S_sordida_30523 | RPA2 | PX148649 | K. Yıldız 0392 | Turkey: Mugla |
| S_sordida_30524 | RPA2 | PX148648 | K. Yıldız 0295-3 | Turkey: Mugla |
| S_ajanensis_7441_643 | RPB2 | AJ634075 | Mikhajlova [? | Russia: Yakutia |
| S_ajanensis_6362_774 | RPB2 | AJ634196 | Magnus Popp 200206 UPS | Russia |
| S_ajanensis_6362_852 | RPB2 | AJ634197 | Magnus Popp 200206 UPS | Russia |
| S_ajanensis_6362_899 | RPB2 | AJ634198 | Magnus Popp 200206 UPS | Russia |
| S_ajanensis_7049_2133 | RPB2 | PX223027 | Anja Rautenberg 68 UPS | Russia: Magadan |
| S_ajanensis_7049_2134 | RPB2 | PX223023 | Anja Rautenberg 68 UPS | Russia: Magadan |
| S_auriculata_14154_5136 | RPB2 | PX223014 | Baden and Franzén 795 Strid | Greece: Korinthos |
| S_cryptoneura_1115_1714 | RPB2 | PX223037 | Bengt Oxelman 1628 GB | Turkey: Antalya |
| S_cryptoneura_1115_1715 | RPB2 | PX223038 | Bengt Oxelman 1628 GB | Turkey: Antalya |
| S_cryptoneura_1115_2310 | RPB2 | PX254881 | Bengt Oxelman 1628 GB | Turkey: Antalya |
| S_cryptoneura_14858_5285 | RPB2 | PX223036 | Bengt Oxelman 2513 GB | Turkey |
| S_davidii_13374_2592 | RPB2 | HM595239 | Frida Eggens 85 GB | China: Sichuan |
| S_davidii_13375_2594 | RPB2 | PX254878 | Frida Eggens 86 GB | China: Sichuan |
| S_ertekinii_1177_264 | RPB2 | AJ296138 | Bengt Oxelman 1691 GB | Turkey: Antalya |
| S_ertekinii_15178_5135 | RPB2 | PX223044 | Zeynep Aydın 36 GB | Turkey: Antalya |
| S_ertekinii_15351_5628 | RPB2 | PX223043 | Zeynep Aydın 31 GB | Turkey: Antalya |
| S_ertekinii_15351_5629 | RPB2 | PX223039 | Zeynep Aydın 31 GB | Turkey: Antalya |
| S_ertekinii_15351_5630 | RPB2 | PX223040 | Zeynep Aydın 31 GB | Turkey: Antalya |
| S_ertekinii_15351_5631 | RPB2 | PX223041 | Zeynep Aydın 31 GB | Turkey: Antalya |
| S_insularis_5509_164 | RPB2 | PX223035 | Greuter 18905 B | Greece: Dodekanisou |
| S_insularis_15372_5287 | RPB2 | PX223032 | Raus 9700 UPS | Greece: Dodekanisou |
| S_joerstadii_7316_1126 | RPB2 | PX254879 | Hedge and Wendelbo W 8992 GB | Afghanistan: Paktiva |
| S_joerstadii_7316_5650 | RPB2 | PX223015 | Hedge and Wendelbo W 8992 GB | Afghanistan: Paktiva |
| S_nemoralis_2465_1552 | RPB2 | PX223016 | Bengt Oxelman 2259 GB | Austria |
| S_odontopetala_14169_5137 | RPB2 | PX223021 | Görk et al. 23817 Strid | Turkey |
| S_salamandra_2410_284 | RPB2 | PX223034 | Bengt Oxelman 2205 GB | Greece: Dodekanisou |
| S_salamandra_15345_5286 | RPB2 | PX223033 | Bengt Oxelman 2541 GB | Greece |
| S_samojedorum_12338_2024 | RPB2 | PX223025 | H. Solstad, R. Elven SUP-04-3871 O | Russia: Sakha Republic (Yakutia) |
| S_samojedorum_12339_2030 | RPB2 | PX223020 | Peter Schönswetter, Andreas Tribsch T601 GB | Russia |
| S_schafta_7615_187 | RPB2 | AJ634088 | Magnus Popp 1053 GB | Cultivated Plants (UPS) |
| S_schafta_7615_2315 | RPB2 | FJ376919 | Magnus Popp 1053 GB | Cultivated Plants (UPS) |
| S_sordida_2411_294 | RPB2 | PX223026 | Bengt Oxelman 2206 GB | Turkey: Mughla |
| S_sordida_14840_5718 | RPB2 | PX223028 | Bengt Oxelman 2495 GB | Turkey: Mughla |
| S_uralensis_7023_864 | RPB2 | PX223012 | Inger Skrede SUP02-38-8 O | Greenland |
| S_uralensis_7587_873 | RPB2 | AJ634204 | Reidar Elven et al. SUP02-1002-2 O | Alaska |
| S_uralensis_7587_937 | RPB2 | AJ634205 | Reidar Elven et al. SUP02-1002-2 O | Alaska |
| S_uralensis_6432_1145 | RPB2 | PX223013 | Lidén 301 UPS | Mongolia |
| S_cryptoneura_30526 | RPB2 | PX223031 | K. Yıldız 0318 | Turkey: Antalya |
| S_cryptoneura_30527 | RPB2 | PX223030 | K. Yıldız 0397-1 | Turkey: Antalya |
| S_cryptoneura_30528 | RPB2 | PX223029 | K. Yıldız 0551 | Turkey: Antalya |
| S_ertekinii_30525 | RPB2 | PX223042 | K. Yıldız 0558 | Turkey: Antalya |
| S_odontopetala_30518 | RPB2 | PX254882 | K. Yıldız 013-2 | Turkey: Kahramanmaraş |
| S_odontopetala_30519 | RPB2 | PX223017 | K. Yıldız 0299-4 | Turkey: Isparta |
| S_odontopetala_30520 | RPB2 | PX254880 | K. Yıldız 0593-2 | Turkey: Uşak |
| S_odontopetala_30521 | RPB2 | PX223019 | K. Yıldız 337-2 | Turkey: Bayburt |
| S_odontopetala_30522 | RPB2 | PX223018 | K. Yıldız 0369-4 | Turkey: Isparta |
| S_sordida_30523 | RPB2 | PX223022 | K. Yıldız 0392 | Turkey: Mugla |
| S_sordida_30524 | RPB2 | PX223024 | K. Yıldız 0295-3 | Turkey: Mugla |
| S_ajanensis_7441_74 | rps16 | LC423915 | Mikhajlova [? | Russia: Yakutia |
| S_ajanensis_6362_842 | rps16 | AJ831763 | Magnus Popp 200206 UPS | Russia |
| S_ajanensis_7049_2041 | rps16 | JF970809 | Anja Rautenberg 68 UPS | Russia: Magadan |
| S_ajanensis_12534_2248 | rps16 | LC423914 | Khokhrjakov LE | Russia |
| S_auriculata_14154_3330 | rps16 | LC423636 | Baden and Franzén 795 Strid | Greece: Korinthos |
| S_cryptoneura_1115_1753 | rps16 | LC423777 | Bengt Oxelman 1628 GB | Turkey: Antalya |
| S_cryptoneura_14858_6105 | rps16 | LC423772 | Bengt Oxelman 2513 GB | Turkey |
| S_cryptoneura_14849_6106 | rps16 | LC423773 | Bengt Oxelman 2504 GB | Turkey |
| S_cryptoneura_17888_6506 | rps16 | LC423668 | Deniz 1573 GB | Turkey |
| S_davidii_13374_2599 | rps16 | FN821271 | Frida Eggens 85 GB | China: Sichuan |
| S_davidii_13375_2601 | rps16 | JF970816 | Frida Eggens 86 GB | China: Sichuan |
| S_ertekinii_1177_79 | rps16 | Z83187 | Bengt Oxelman 1691 GB | Turkey: Antalya |
| S_ertekinii_15351_6074 | rps16 | LC423679 | Zeynep Aydın 31 GB | Turkey: Antalya |
| S_ertekinii_15352_6075 | rps16 | LC423771 | Zeynep Aydın 28 GB | Turkey: Antalya |
| S_ertekinii_15178_6079 | rps16 | LC423770 | Zeynep Aydın 36 GB | Turkey: Antalya |
| S_insularis_5509_110 | rps16 | LC423776 | Greuter 18905 B | Greece: Dodekanisou |
| S_insularis_15372_6108 | rps16 | LC423699 | Raus 9700 UPS | Greece: Dodekanisou |
| S_italica_15252_6598 | rps16 | LC423805 | Zeynep Aydın 49 GB | Turkey |
| S_italica_17755_6616 | rps16 | LC423702 | Bengt Oxelman 2619 GB | Turkey |
| S_italica_18998_7183 | rps16 | LC424015 | Eslami TUH | Iran: Mazandaran |
| S_joerstadii_7316_973 | rps16 | LC423704 | Hedge and Wendelbo W 8992 GB | Afghanistan: Paktiva |
| S_nemoralis_2465_1541 | rps16 | EF061389 | Bengt Oxelman 2259 GB | Austria |
| S_odontopetala_14169_3327 | rps16 | LC423764 | Görk et al. 23817 Strid | Turkey |
| S_odontopetala_14169_6113 | rps16 | LC423726 | Görk et al. 23817 Strid | Turkey |
| S_odontopetala_15381_6369 | rps16 | LC423763 | Bengt Oxelman 2612 GB | Iran |
| S_odontopetala_17765_6614 | rps16 | LC423765 | Bengt Oxelman 2631 GB | Turkey |
| S_odontopetala_19033_7152 | rps16 | LC423984 | A. Gholipour SPNH | Iran: Mazandaran |
| S_salamandra_6058_1780 | rps16 | LC423775 | Carlström 2308 LD | Greece: Dodekanisou |
| S_salamandra_15345_6107 | rps16 | LC423774 | Bengt Oxelman 2541 GB | Greece |
| S_samojedorum_12338_2028 | rps16 | JF970807 | H. Solstad, R. Elven SUP-04-3871 O | Russia: Sakha Republic (Yakutia) |
| S_samojedorum_12339_2033 | rps16 | JF970808 | Peter Schönswetter, Andreas Tribsch T601 GB | Russia |
| S_schafta_2474_1160 | rps16 | Z83194 | Bengt Oxelman 2264 GB | Cultivated Plants (Iran?) |
| S_schafta_19029_7170 | rps16 | LC424002 | SPNH | Iran: Mazandaran |
| S_seoulensis_7812_1988 | rps16 | JF970806 | Hong and Han 13420001 UPS | Korea |
| S_sordida_2411_1123 | rps16 | Z83186 | Bengt Oxelman 2206 GB | Turkey: Mughla |
| S_sordida_7824_1773 | rps16 | LC423769 | Bengt Oxelman 2206 GB | Turkey: Mughla |
| S_sordida_14840_6102 | rps16 | LC423766 | Bengt Oxelman 2495 GB | Turkey: Mughla |
| S_sordida_14838_6103 | rps16 | LC423768 | Bengt Oxelman 2493 GB | Turkey: Mughla |
| S_sordida_14835_6104 | rps16 | LC423767 | Bengt Oxelman 2492 GB | Turkey: Mughla |
| S_uralensis_7614_123 | rps16 | LC423913 | Magnus Popp 1052 UPS | Cultivated Plants |
| S_uralensis_6432_816 | rps16 | AJ831769 | Lidén 301 UPS | Mongolia |
| S_uralensis_6364_843 | rps16 | AJ831767 | Fredskild and Dalgaard 87837 C | Greenland |
| S_uralensis_7023_844 | rps16 | AJ831768 | Inger Skrede SUP02-38-8 O | Greenland |
| S_uralensis_7587_847 | rps16 | AJ831765 | Reidar Elven et al. SUP02-1002-2 O | Alaska |
| S_uralensis_14594_4635 | rps16 | JF970847 | Lidén 24-301 GB | Mongolia |
| S_cryptoneura_30526 | rps16 | PX216448 | K. Yıldız 0318 | Turkey: Antalya |
| S_cryptoneura_30527 | rps16 | PX216449 | K. Yıldız 0397-1 | Turkey: Antalya |
| S_cryptoneura_30528 | rps16 | PX216450 | K. Yıldız 0551 | Turkey: Antalya |
| S_ertekinii_30525 | rps16 | PX216447 | K. Yıldız 0558 | Turkey: Antalya |
| S_odontopetala_30518 | rps16 | PX216440 | K. Yıldız 013-2 | Turkey: Kahramanmaraş |
| S_odontopetala_30519 | rps16 | PX216441 | K. Yıldız 0299-4 | Turkey: Isparta |
| S_odontopetala_30520 | rps16 | PX216442 | K. Yıldız 0593-2 | Turkey: Uşak |
| S_odontopetala_30521 | rps16 | PX216443 | K. Yıldız 337-2 | Turkey: Bayburt |
| S_odontopetala_30522 | rps16 | PX216444 | K. Yıldız 0369-4 | Turkey: Isparta |
| S_sordida_30523 | rps16 | PX216445 | K. Yıldız 0392 | Turkey: Mugla |
| S_sordida_30524 | rps16 | PX216446 | K. Yıldız 0295-3 | Turkey: Mugla |

* The rows highlighted in red colour present the newly generated sequences

**Table S2**. Details of the primer sequences and PCR programs

|  | Rps16 | RPA2 | | RPB2 | | EST04 | | ITS |
| --- | --- | --- | --- | --- | --- | --- | --- | --- |
| Forward primer  5’ ------3’ | ***rpsF:*** GTGGTAGAAAGCAACGTGCGACTT | | ***RPA2FP:*** GCCGTTTTCWGAGATAACTGGGATGCGT | | ***f7327:*** CCATCYCGTATGACAATCGGYCAGCTT | ***silene_ est4-F:*** CTGCTGTTGGACAGGATTGTG | ***ITS4:*** TCCTCCGCTTATTGATATGC | |
| Reverse primer  5’ ------3’ | ***rpsR2R:*** TCGGGATCGAACATCAATTGCAAC | | ***RPA2RP:*** GRTAATAAACAGGYCCAATAAAGATCTC | | ***r7586:*** CCCMGTGTGACCATTGTACATTGTCT | ***silene_ est4-R:*** CATCTCCACCAGTCTCAACACC | ***ITS5:***  GGAAGTAAAAGTCGTAACAAGG | |
| PCR Program | 95°C 4’ (95°C 30” + 57°C 30” + 72°C 60”)  X 35 + 72°C 5’ + 4°C ∞ | | 97°C 2,5’ (97°C 15” + 62°C 25” + 72°C 60”)  X 35 + 72°C 5’ + 4°C ∞ | | 97°C 2,5’ (97°C 15” + 62°C 25” + 72°C 60”) X 35 + 72°C 5’ + 4°C ∞ | 94°C 3’ (94°C 30” + 60°C 30” + 72°C 60”) X 42 + 72°C 5’ + 4°C ∞ | 95°C 4’ (95°C 30” + 57°C 30” + 72°C 60”) X 35 /+ 72°C 5’ + 4°C ∞ | |
| Sequencing Primers | *rpsF/rpsR2R* | | *RPA2FP/RPA2RP* | | *f7327/r7586* | *silene_ est4F/silene_ est4-R* | *ITS4/ITS5* | |

**Table S3**. Parsimony analysis statistics for the five loci used in phylogenetic reconstruction

| **Sequenced regions** | **Rps16** | **ITS** | **NRPA2** | **NRPB2** | **EST04** |
| --- | --- | --- | --- | --- | --- |
| **Number of samples** | 48 | 49 | 47 | 46 | 26 |
| **Aligned matrix length [bp]** | 885 | 805 | 860 | 972 | 732 |
| **Constant characters [bp]** | 757 | 664 | 699 | 829 | 650 |
| **Parsimony-uninformative [bp]** | 48 | 29 | 48 | 43 | 12 |
| **Parsimony-informative characters [bp]** | 80 | 112 | 113 | 100 | 70 |
| **Consistency index (CI)** | 0.891 | 0.684 | 0.918 | 0.897 | 0.915 |
| **Retention index (RI)** | 0.972 | 0.906 | 0.971 | 0.972 | 0.978 |
| **Homoplasy index (HI)** | 0.109 | 0.316 | 0.082 | 0.103 | 0.085 |
| **Rescaled consistency index (RC)** | 0.866 | 0.620 | 0.892 | 0.872 | 0.895 |
| **Substitution model** | K81uf+G | GTR+I+G | HKY+G | K81uf +I | HKY+I |

**Figure S1.** Maximum-clade-credibility gene trees inferred from five loci: (A) ***rps16***, (B) ***ITS***, (C) ***RPA2***, (D) ***RPB2***, and (E) ***EST04***, using the StarBEAST3 module in the BEAST2 package. Numbers above branches represent Bayesian posterior probabilities(PP) for major clades. Tip labels are coloured according to sectional assignments within the genus ***Silene.*** The scale bar indicates substitutions per site. Numbers next to taxon names correspond to unique specimen identification numbers in the Sileneae BoxTax database ([*http: //www.sileneae.info*](http://www.sileneae.info)). Figures were generated using FigTree v1.4.4 and Inkscape 1.1.1.
