# Supplementary material for: ﻿Phylogenetic analysis suggests early divergence followed by convergent morphological evolution in the Silene sections Odontopetalae and Sordidae (Caryophyllaceae)
Source: PhytoKeys. 2025 Oct 24;265:123–45. doi: 10.3897/phytokeys.265.165998 (PMC12579334; doi:10.3897/phytokeys.265.165998)
Supplement: Supplementary material 3 — Quartet sampling (QS) values obtained from ASTRAL-III [file phytokeys-265-123_article-165998__-s003.docx]

**Supplementary Table 4.** Shows the QS parameters for the Phylogenetic relationships under the strict-clock model

**Table :4**

| Branch | QS (q1​) | QS (q2​,q3​) | F (f1​) | F (f2​,f3​) | PP (pp1​) | PP (pp2​,pp3​) | QC | EN |
| --- | --- | --- | --- | --- | --- | --- | --- | --- |
| ajanensis | 0.385 | 0.307, 0.307 | 0.385 | 0.307, 0.307 | 0.355 | 0.323, 0.323 | 181440 | 1 |
| samojedorum | 0.38 | 0.310, 0.310 | 0.38 | 0.310, 0.310 | 0.352 | 0.324, 0.324 | 103680 | 1 |
| Internal Branch 1 | 0.883 | 0.002, 0.114 | 3.533 | 0.010, 0.457 | 0.945 | 0.024, 0.031 | 504900 | 4 |
| davidii | 1 | 0.000, 0.000 | 1 | 0.000, 0.000 | 0.667 | 0.167, 0.167 | 90792 | 1 |
| uralensis | 0.846 | 0.077, 0.077 | 0.846 | 0.077, 0.077 | 0.582 | 0.209, 0.209 | 136188 | 1 |
| Internal Branch 2 | 0.256 | 0.417, 0.327 | 1.026 | 1.667, 1.308 | 0.259 | 0.424, 0.317 | 477360 | 4 |
| Internal Branch 3 | 0.333 | 0.048, 0.619 | 1 | 0.143, 1.857 | 0.249 | 0.135, 0.616 | 264528 | 3 |
| seoulensis | NaN | NaN, NaN | 0 | 0.000, 0.000 | 0.333 | 0.333, 0.333 | 23046 | 0 |
| Internal Branch 4 | 0.974 | 0.026, 0.000 | 2.923 | 0.077, 0.000 | 0.942 | 0.030, 0.029 | 929016 | 3 |
| auriculata | NaN | NaN, NaN | 0 | 0.000, 0.000 | 0.333 | 0.333, 0.333 | 21156 | 0 |
| joerstadii | 1 | 0.000, 0.000 | 0.625 | 0.000, 0.000 | 0.554 | 0.223, 0.223 | 161700 | 0.625 |
| odontopetala | 0.446 | 0.277, 0.277 | 0.446 | 0.277, 0.277 | 0.38 | 0.310, 0.310 | 739200 | 1 |
| Internal Branch 5 | 0.672 | 0.140, 0.188 | 2.015 | 0.420, 0.565 | 0.693 | 0.146, 0.161 | 136224 | 3 |
| Internal Branch 6 | 0.731 | 0.269, 0.000 | 2.194 | 0.806, 0.000 | 0.747 | 0.159, 0.093 | 436896 | 3 |
| sordida | 1 | 0.000, 0.000 | 1 | 0.000, 0.000 | 0.667 | 0.167, 0.167 | 1797312 | 1 |
| Internal Branch 7 | 0.108 | 0.482, 0.410 | 0.431 | 1.929, 1.640 | 0.159 | 0.476, 0.365 | 5122656 | 4 |
| Internal Branch 8 | 0.498 | 0.502, 0.000 | 1.991 | 2.009, 0.000 | 0.44 | 0.448, 0.111 | 5824336 | 4 |
| cryptoneura | 0.348 | 0.326, 0.326 | 0.348 | 0.326, 0.326 | 0.339 | 0.330, 0.330 | 1482048 | 1 |
| insularis | 0.393 | 0.304, 0.304 | 0.393 | 0.304, 0.304 | 0.358 | 0.321, 0.321 | 55440 | 1 |
| salamandra | 0.662 | 0.169, 0.169 | 0.595 | 0.152, 0.152 | 0.471 | 0.265, 0.265 | 71280 | 0.9 |
| Internal Branch 9 | 0.604 | 0.208, 0.188 | 2.417 | 0.833, 0.750 | 0.675 | 0.167, 0.158 | 424960 | 4 |
| Internal Branch 10 | 1 | 0.000, 0.000 | 5 | 0.000, 0.000 | 0.995 | 0.003, 0.003 | 2096640 | 5 |
| ertekinii | 1 | 0.000, 0.000 | 1 | 0.000, 0.000 | 0.667 | 0.167, 0.167 | 2275000 | 1 |
| Internal Branch 11 | 1 | 0.000, 0.000 | 5 | 0.000, 0.000 | 0.995 | 0.003, 0.003 | 3498300 | 5 |
| Internal Branch 12 | 0.88 | 0.120, 0.000 | 3.52 | 0.480, 0.000 | 0.943 | 0.032, 0.025 | 1155960 | 4 |
| italica | 1 | 0.000, 0.000 | 0.857 | 0.000, 0.000 | 0.626 | 0.187, 0.187 | 25578 | 0.857 |
| nemoralis | 0 | 0.500, 0.500 | 0 | 0.250, 0.250 | 0.264 | 0.368, 0.368 | 21315 | 0.5 |
| Internal Branch 13 | 1 | 0.000, 0.000 | 3 | 0.000, 0.000 | 0.952 | 0.024, 0.024 | 81060 | 3 |
| schafta | 1 | 0.000, 0.000 | 0.7 | 0.000, 0.000 | 0.578 | 0.211, 0.211 | 112905 | 0.7 |
| Internal Branch 14 | 0.88 | 0.120, 0.000 | 3.52 | 0.480, 0.000 | 0.943 | 0.032, 0.025 | 1155960 | 4 |

**Reference-Tree under strict clock Model:**

((((((ajanensis:0.0,samojedorum:0.0)'[q1=0.8833333333333333;q2=0.0024509803921568627;q3=0.11421568627450981;f1=3.533333333333333;f2=0.00980392156862745;f3=0.45686274509803926;pp1=0.9450666005074844;pp2=0.024370717234899444;pp3=0.03056268225761618;QC=504900;EN=4.0]':0.8209805520698302,(davidii:0.2876820724517809,uralensis:0.1445812288111077)'[q1=0.2564102564102564;q2=0.41666666666666663;q3=0.3269230769230769;f1=1.0256410256410255;f2=1.6666666666666665;f3=1.3076923076923077;pp1=0.25885653041040074;pp2=0.42442826208333384;pp3=0.3167152075062654;QC=477360;EN=4.0]':0.0)'[q1=0.3333333333333333;q2=0.047619047619047616;q3=0.6190476190476191;f1=1.0;f2=0.14285714285714285;f3=1.8571428571428572;pp1=0.24896947863438101;pp2=0.1351884790386034;pp3=0.6158420423270157;QC=264528;EN=3.0]':0.0,seoulensis:0.0)'[q1=0.9743589743589745;q2=0.025641025641025644;q3=0.0;f1=2.9230769230769234;f2=0.07692307692307693;f3=0.0;pp1=0.9416816689629325;pp2=0.029798390063547564;pp3=0.028519940973519852;QC=929016;EN=3.0]':0.9067212808580046,((auriculata:0.0,(joerstadii:0.08004270767353637,odontopetala:0.0)'[q1=0.6716716716716716;q2=0.14014014014014015;q3=0.18818818818818817;f1=2.015015015015015;f2=0.42042042042042044;f3=0.5645645645645645;pp1=0.6926585845040305;pp2=0.1461028965605739;pp3=0.16123851893539556;QC=136224;EN=3.0]':0.2952179031404838)'[q1=0.7314880952380953;q2=0.26851190476190473;q3=0.0;f1=2.194464285714286;f2=0.8055357142857142;f3=0.0;pp1=0.7472306165236702;pp2=0.1593611742058037;pp3=0.09340820927052598;QC=436896;EN=3.0]':0.3899719106435679,sordida:0.2876820724517809)'[q1=0.10779220779220779;q2=0.48214285714285715;q3=0.4100649350649351;f1=0.43116883116883115;f2=1.9285714285714286;f3=1.6402597402597403;pp1=0.15888046875819292;pp2=0.4762971424557944;pp3=0.36482238878601264;QC=5122656;EN=4.0]':0.0)'[q1=0.49776785714285715;q2=0.5022321428571428;q3=0.0;f1=1.9910714285714286;f2=2.008928571428571;f3=0.0;pp1=0.44041794078588525;pp2=0.44847677637240907;pp3=0.11110528284170561;QC=5824336;EN=4.0]':0.1023887452686688,((cryptoneura:0.0,(insularis:0.0,salamandra:0.0)'[q1=0.6041666666666666;q2=0.20833333333333331;q3=0.1875;f1=2.4166666666666665;f2=0.8333333333333333;f3=0.75;pp1=0.6752992766803002;pp2=0.16653042581954292;pp3=0.15817029750015676;QC=424960;EN=4.0]':0.25489224962878987)'[q1=1.0;q2=0.0;q3=0.0;f1=5.0;f2=0.0;f3=0.0;pp1=0.9945355191256833;pp2=0.002732240437158474;pp3=0.002732240437158474;QC=2096640;EN=5.0]':1.3862943611198908,ertekinii:0.2876820724517809)'[q1=1.0;q2=0.0;q3=0.0;f1=5.0;f2=0.0;f3=0.0;pp1=0.9945355191256833;pp2=0.002732240437158474;pp3=0.002732240437158474;QC=3498300;EN=5.0]':1.3862943611198908)'[q1=0.8799603174603174;q2=0.12003968253968253;q3=0.0;f1=3.5198412698412698;f2=0.4801587301587301;f3=0.0;pp1=0.9432945420490347;pp2=0.031791608914141505;pp3=0.02491384903682375;QC=1155960;EN=4.0]':0.8118234721935436,((italica:0.2135741002980589,nemoralis:0.0)'[q1=1.0;q2=0.0;q3=0.0;f1=3.0;f2=0.0;f3=0.0;pp1=0.9523809523809523;pp2=0.023809523809523822;pp3=0.023809523809523822;QC=81060;EN=3.0]':0.9808292530117262,schafta:0.12516314295400605)'[q1=0.8799603174603174;q2=0.12003968253968253;q3=0.0;f1=3.5198412698412698;f2=0.4801587301587301;f3=0.0;pp1=0.9432945420490347;pp2=0.031791608914141505;pp3=0.02491384903682375;QC=1155960;EN=4.0]':0.8118234721935436);

**Supplementary Table 5**. Shows the QS parameters for the phylogenetic relationships under the relax-clock model

**Table 5**

| Branch | QS (q1) | QS (q2, q3) | F (f1) | F (f2, f3) | PP (pp1) | PP (pp2, pp3) | QC | EN |
| --- | --- | --- | --- | --- | --- | --- | --- | --- |
| S_ajanensis | 0.385 | 0.307, 0.307 | 0.385 | 0.307, 0.307 | 0.355 | 0.323, 0.323 | 181440 | 1 |
| S_samojedorum | 0.38 | 0.310, 0.310 | 0.38 | 0.310, 0.310 | 0.352 | 0.324, 0.324 | 103680 | 1 |
| Internal Branch 1 | 0.883 | 0.002, 0.114 | 3.533 | 0.010, 0.457 | 0.945 | 0.024, 0.031 | 504900 | 4 |
| S_davidii | 1 | 0.000, 0.000 | 1 | 0.000, 0.000 | 0.667 | 0.167, 0.167 | 90792 | 1 |
| S_uralensis | 0.846 | 0.077, 0.077 | 0.846 | 0.077, 0.077 | 0.582 | 0.209, 0.209 | 136188 | 1 |
| Internal Branch 2 | 0.256 | 0.417, 0.327 | 1.026 | 1.667, 1.308 | 0.259 | 0.424, 0.317 | 477360 | 4 |
| Internal Branch 3 | 0.333 | 0.048, 0.619 | 1 | 0.143, 1.857 | 0.249 | 0.135, 0.616 | 264528 | 3 |
| S_seoulensis | NaN | NaN, NaN | 0 | 0.000, 0.000 | 0.333 | 0.333, 0.333 | 23046 | 0 |
| Internal Branch 4 | 0.974 | 0.026, 0.000 | 2.923 | 0.077, 0.000 | 0.942 | 0.030, 0.029 | 457056 | 3 |
| S_auriculata | NaN | NaN, NaN | 0 | 0.000, 0.000 | 0.333 | 0.333, 0.333 | 21156 | 0 |
| S_joerstadii | 1 | 0.000, 0.000 | 0.625 | 0.000, 0.000 | 0.554 | 0.223, 0.223 | 161700 | 0.625 |
| S2_odontopetala | 0.446 | 0.277, 0.277 | 0.446 | 0.277, 0.277 | 0.38 | 0.310, 0.310 | 739200 | 1 |
| Internal Branch 5 | 0.672 | 0.140, 0.188 | 2.015 | 0.420, 0.565 | 0.693 | 0.146, 0.161 | 136224 | 3 |
| Internal Branch 6 | 0.596 | 0.343, 0.061 | 1.787 | 1.030, 0.183 | 0.588 | 0.267, 0.145 | 885600 | 3 |
| S10_cryptoneura | 0.348 | 0.326, 0.326 | 0.348 | 0.326, 0.326 | 0.339 | 0.330, 0.330 | 1482048 | 1 |
| S_insularis | 0.393 | 0.304, 0.304 | 0.393 | 0.304, 0.304 | 0.358 | 0.321, 0.321 | 55440 | 1 |
| S_salamandra | 0.662 | 0.169, 0.169 | 0.595 | 0.152, 0.152 | 0.471 | 0.265, 0.265 | 71280 | 0.9 |
| Internal Branch 7 | 0.604 | 0.208, 0.188 | 2.417 | 0.833, 0.750 | 0.675 | 0.167, 0.158 | 424960 | 4 |
| Internal Branch 8 | 1 | 0.000, 0.000 | 5 | 0.000, 0.000 | 0.995 | 0.003, 0.003 | 2096640 | 5 |
| S_ertekinii | 1 | 0.000, 0.000 | 1 | 0.000, 0.000 | 0.667 | 0.167, 0.167 | 2275000 | 1 |
| Internal Branch 9 | 1 | 0.000, 0.000 | 5 | 0.000, 0.000 | 0.995 | 0.003, 0.003 | 3619200 | 5 |
| S6_sordida | 1 | 0.000, 0.000 | 1 | 0.000, 0.000 | 0.667 | 0.167, 0.167 | 2433216 | 1 |
| Internal Branch 10 | 0.4 | 0.146, 0.454 | 2 | 0.728, 2.272 | 0.37 | 0.165, 0.465 | 5778432 | 5 |
| Internal Branch 11 | 0.495 | 0.116, 0.389 | 1.981 | 0.462, 1.557 | 0.5 | 0.161, 0.339 | 4958800 | 4 |
| Internal Branch 12 | 0.74 | 0.000, 0.260 | 2.961 | 0.000, 1.039 | 0.83 | 0.061, 0.109 | 917280 | 4 |
| S_schafta | 1 | 0.000, 0.000 | 0.7 | 0.000, 0.000 | 0.578 | 0.211, 0.211 | 112905 | 0.7 |
| S_italica | 1 | 0.000, 0.000 | 0.857 | 0.000, 0.000 | 0.626 | 0.187, 0.187 | 25578 | 0.857 |
| S_nemoralis | 0 | 0.500, 0.500 | 0 | 0.250, 0.250 | 0.264 | 0.368, 0.368 | 21315 | 0.5 |
| Internal Branch 13 | 1 | 0.000, 0.000 | 3 | 0.000, 0.000 | 0.952 | 0.024, 0.024 | 81060 | 3 |
| Internal Branch 14 | 1 | 0.000, 0.000 | 3 | 0.000, 0.000 | 0.952 | 0.024, 0.024 | 81060 | 3 |

**Reference-Tree under relax clock Model**

((((((ajanensis:0.0,samojedorum:0.0)'[q1=0.8833333333333333;q2=0.0024509803921568627;q3=0.11421568627450981;f1=3.533333333333333;f2=0.00980392156862745;f3=0.45686274509803926;pp1=0.9450666005074844;pp2=0.024370717234899444;pp3=0.03056268225761618;QC=504900;EN=4.0]':0.8209805520698302,(davidii:0.2876820724517809,uralensis:0.1445812288111077)'[q1=0.2564102564102564;q2=0.41666666666666663;q3=0.3269230769230769;f1=1.0256410256410255;f2=1.6666666666666665;f3=1.3076923076923077;pp1=0.25885653041040074;pp2=0.42442826208333384;pp3=0.3167152075062654;QC=477360;EN=4.0]':0.0)'[q1=0.3333333333333333;q2=0.047619047619047616;q3=0.6190476190476191;f1=1.0;f2=0.14285714285714285;f3=1.8571428571428572;pp1=0.24896947863438101;pp2=0.1351884790386034;pp3=0.6158420423270157;QC=264528;EN=3.0]':0.0,seoulensis:0.0)'[q1=0.9743589743589745;q2=0.025641025641025644;q3=0.0;f1=2.9230769230769234;f2=0.07692307692307693;f3=0.0;pp1=0.9416816689629325;pp2=0.029798390063547564;pp3=0.028519940973519852;QC=457056;EN=3.0]':0.9067212808580046,((auriculata:0.0,(joerstadii:0.08004270767353637,odontopetala:0.0)'[q1=0.6716716716716716;q2=0.14014014014014015;q3=0.18818818818818817;f1=2.015015015015015;f2=0.42042042042042044;f3=0.5645645645645645;pp1=0.6926585845040305;pp2=0.1461028965605739;pp3=0.16123851893539556;QC=136224;EN=3.0]':0.2952179031404838)'[q1=0.5955555555555555;q2=0.3433333333333333;q3=0.06111111111111111;f1=1.7866666666666666;f2=1.03;f3=0.18333333333333332;pp1=0.588245189391593;pp2=0.26700298554734425;pp3=0.14475182506106282;QC=885600;EN=3.0]':0.18632957819149337,(((cryptoneura:0.0,(insularis:0.0,salamandra:0.0)'[q1=0.6041666666666666;q2=0.20833333333333331;q3=0.1875;f1=2.4166666666666665;f2=0.8333333333333333;f3=0.75;pp1=0.6752992766803002;pp2=0.16653042581954292;pp3=0.15817029750015676;QC=424960;EN=4.0]':0.25489224962878987)'[q1=1.0;q2=0.0;q3=0.0;f1=5.0;f2=0.0;f3=0.0;pp1=0.9945355191256833;pp2=0.002732240437158474;pp3=0.002732240437158474;QC=2096640;EN=5.0]':1.3862943611198908,ertekinii:0.2876820724517809)'[q1=1.0;q2=0.0;q3=0.0;f1=5.0;f2=0.0;f3=0.0;pp1=0.9945355191256833;pp2=0.002732240437158474;pp3=0.002732240437158474;QC=3619200;EN=5.0]':1.3862943611198908,sordida:0.2876820724517809)'[q1=0.4;q2=0.14555555555555558;q3=0.45444444444444443;f1=2.0;f2=0.7277777777777779;f3=2.272222222222222;pp1=0.3699993627480769;pp2=0.16549408656413617;pp3=0.46450655068778685;QC=5778432;EN=5.0]':0.0)'[q1=0.4951298701298701;q2=0.11553030303030304;q3=0.3893398268398268;f1=1.9805194805194803;f2=0.46212121212121215;f3=1.5573593073593073;pp1=0.5000452739772017;pp2=0.16109062303149257;pp3=0.3388641029913058;QC=4958800;EN=4.0]':0.09888800115220878)'[q1=0.7402073732718895;q2=0.0;q3=0.2597926267281106;f1=2.960829493087558;f2=0.0;f3=1.0391705069124424;pp1=0.8296216719428586;pp2=0.06091797619957551;pp3=0.10946035185756589;QC=917280;EN=4.0]':0.4914296934184115,schafta:0.12516314295400605)'[q1=1.0;q2=0.0;q3=0.0;f1=3.0;f2=0.0;f3=0.0;pp1=0.9523809523809523;pp2=0.023809523809523822;pp3=0.023809523809523822;QC=81060;EN=3.0]':0.9808292530117262,(italica:0.2135741002980589,nemoralis:0.0)'[q1=1.0;q2=0.0;q3=0.0;f1=3.0;f2=0.0;f3=0.0;pp1=0.9523809523809523;pp2=0.023809523809523822;pp3=0.023809523809523822;QC=81060;EN=3.0]':0.9808292530117262);

**QS:** (q1, q2, q3): Quartet Support (normalized). These three values show the normalized quartet score for the three possible unrooted topologies around the branch: q1 for the main topology (the one shown in the species tree), q2 for the first alternative, and q3 for the second alternative.

**F:** (f1, f2, f3) : Quartet Frequencies (Total Support). These values show the total number of quartet trees in all the gene trees that support the main topology (f1), the first alternative (f2), and the second alternative (f3).

**PP:** (pp1, pp2, pp3): Local Posterior Probabilities. These values represent the local posterior probabilities for the main topology (pp1), the first alternative (pp2), and the second alternative (pp3). They provide a measure of confidence in the topology of the branch.

**QC:** Quartet Count or Total Informative Gene Trees. This value is the total number of gene trees that had any useful information about this branch. It represents the total number of genes that had at least one species from each of the four groups relevant to the quartet topology.

**EN:** Effective Number (or Number of Gene Trees). In the context of ASTRAL output, it relates to the number of gene trees used in the calculation of the quartet support.

ference Tree under strict clockModel
