# Supplementary figures and images for: ﻿Phylogenetic analysis suggests early divergence followed by convergent morphological evolution in the Silene sections Odontopetalae and Sordidae (Caryophyllaceae)
Source: PhytoKeys. 2025 Oct 24;265:123–45. doi: 10.3897/phytokeys.265.165998 (PMC12579334; doi:10.3897/phytokeys.265.165998)

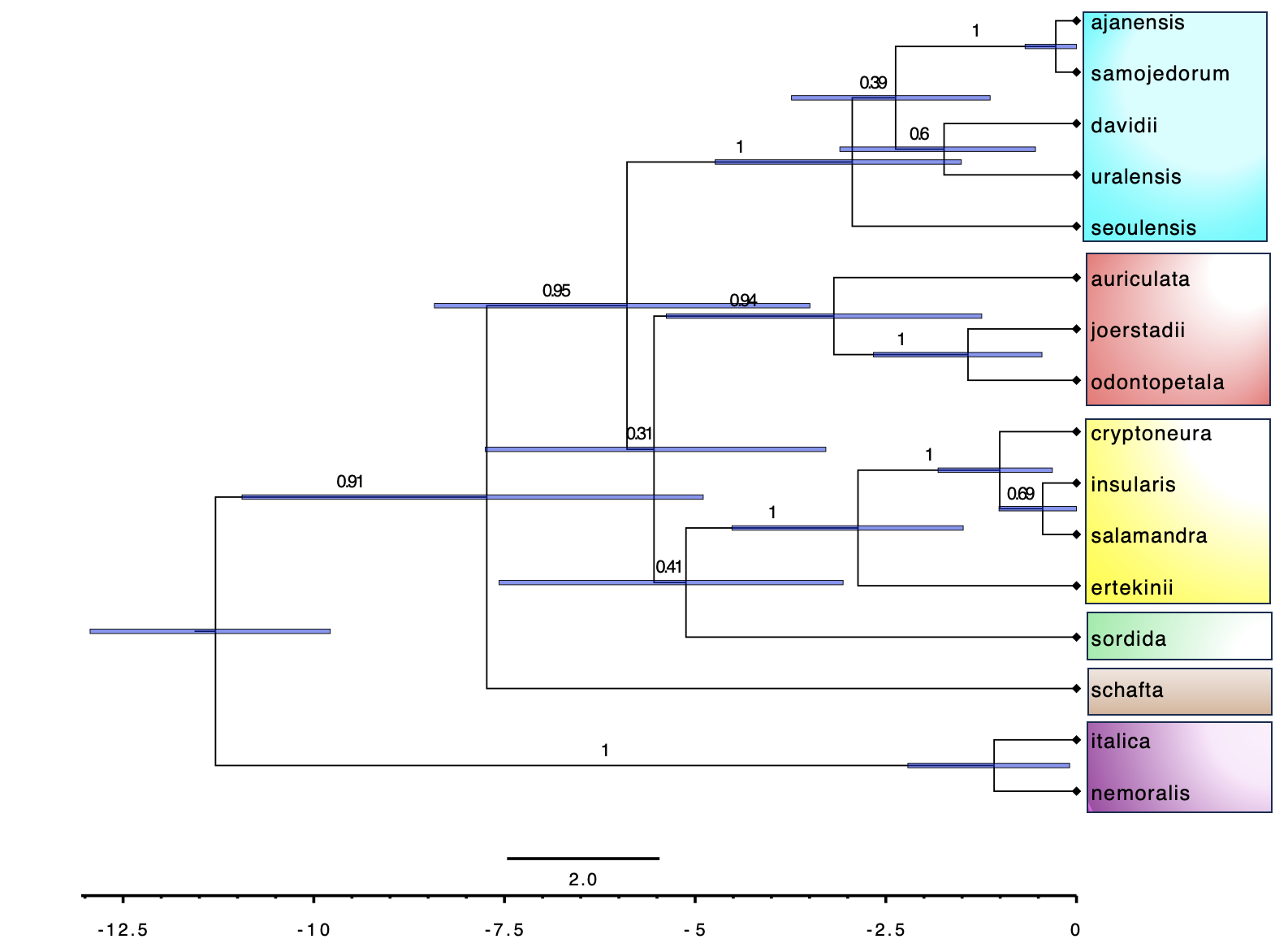

Supplement: Supplementary material 4 — Species phylogeny inferred under a relaxed clock model [file phytokeys-265-123_article-165998__-s004.png]
